# Not All Those Who Wander Are Lost: Insights Into Movement Pattern of the Great Indian Bustard in the Deccan Landscape of India

**DOI:** 10.1002/ece3.71742

**Published:** 2025-07-06

**Authors:** Shaheer Khan, Vaijayanti Vijayaraghavan, Gautam Talukdar, R. Suresh Kumar, Bilal Habib

**Affiliations:** ^1^ Wildlife Institute of India Chandrabani Dehradun India

**Keywords:** cluster analysis, conservation, endangered, movement ecology, utilization distribution

## Abstract

Understanding animal movement patterns, such as daily foraging, seasonal migration, dispersal and other movement behaviours, is essential for effectively managing and conserving species. The Great Indian Bustard ( *Ardeotis nigriceps* ), a critically endangered bird, is thought to move opportunistically in response to environmental changes, particularly rainfall events, which influence food availability and habitat productivity. This research represents the first fine‐scale analysis of the movement behaviour of this species. In this study, we investigated the daily movements, seasonal movement variations and utilization distributions of a sub‐adult Great Indian Bustard. Over the study period, a sub‐adult travelled a total distance of 2208.62 km, with an hourly movement of 0.33 km/h (range: 0–10.4 km/h) and an average daily movement of 5.39 km. The highest daily displacement was found in summer (6.17 km/day; range: 0.26–38.99), followed by winter (5.39 km/day; range: 0.24–28.12) and the lowest was in post‐monsoon (3.15 km/day; range: 0.17–11.49). The bird consistently followed a Lévy flight movement pattern, with a scaling exponent (*μ*) of 2.1, both overall and across different seasons. The core areas of use (50% Kernel Density Estimates; KDE) covered 2633.07 km^2^, forming four distinct clusters, while the extensive utilization area (95% KDE) covered 12,080.78 km^2^. We identified 10 movement clusters within different regions of Maharashtra and Karnataka, which were predominantly composed of open lands, followed by Kharif crop fields and mixed crops. The mean Normalized Difference Vegetation Index (NDVI) value for these clusters was 0.31, ranging from 0.16 to 0.43, emphasizing the significance of specific vegetation cover that offers both protection and food. This study provides baseline information on the movement ecology of the Great Indian Bustard and offers valuable insights for developing more effective conservation and management strategies for this species.

## Graphical abstract

This study presented a sub‐adult Great Indian Bustard's movements over 2208.62 km, revealing significant seasonal variation. It identifies 10 key movement clusters, with core areas covering 2633.07 km^2^ and extensive areas 12,080.78 km^2^, mostly in open lands and crop fields. These insights are crucial for effective conservation strategies for this critically endangered species.

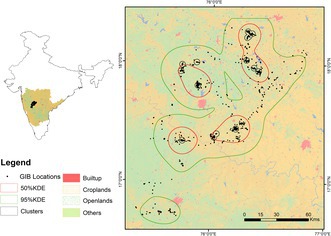

## 1 Introduction

The animal movement centers on many critical ecological processes and helps understand animal behaviour, association and relation to the surroundings (McClintock et al. 2014). Understanding how, why and when animals move, disperse and migrate is fundamental to effective management and species conservation (Jacoby et al. 2012). Moreover, understanding the movement of the species exploiting or sharing a human‐dominated landscape is crucial, as they face comparatively high anthropogenic effects. Animals show various movement patterns of daily foraging, seasonal migration, dispersal and unpredictable nomadic movements (Mueller and Fagan 2008; Nathan et al. 2008). Such animal movements highlight the importance of landscape‐level approaches for management and conservation. Understanding the movements using advanced telemetry methods can help identify essential or critical areas for animal conservation (Wikelski et al. 2007; Habib et al. 2014; Jacoby and Freeman 2016; Moll et al. 2021). Furthermore, comprehensive data can provide insights into behavioural patterns and associated landscape features, aiding in understanding the species and developing effective conservation management strategies (Burdett et al. 2007; Cadahía et al. 2007; Kessler et al. 2013; Laporte et al. 2010; Miller et al. 2010).

**FIGURE 1:**
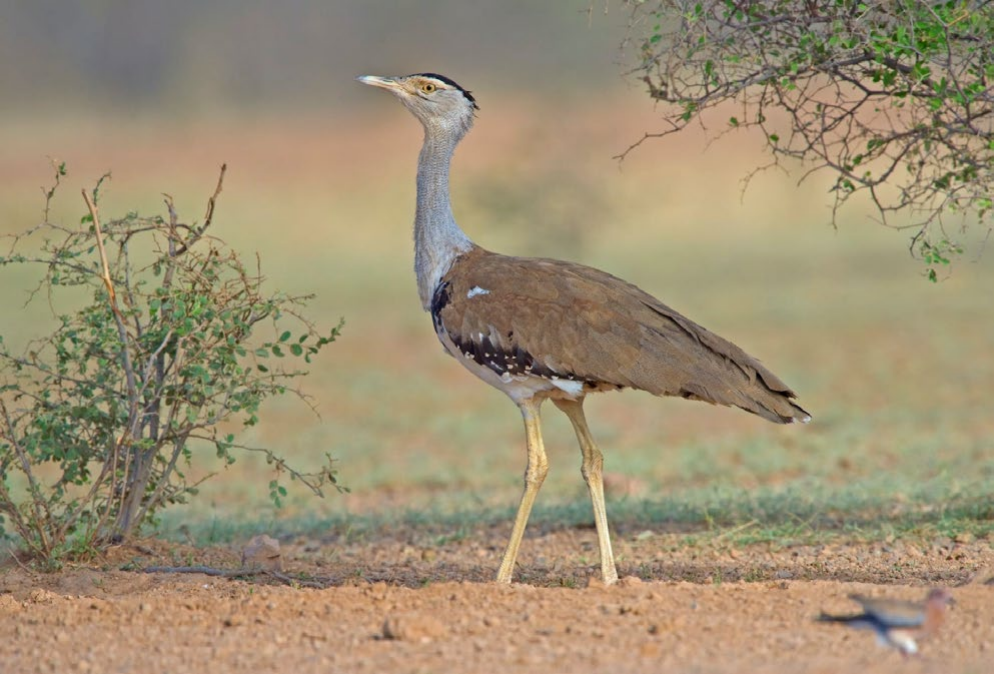
The Great Indian Bustard ( *Ardeotis nigriceps* ), a critically endangered grassland species characterized by its tall stature, long legs, and distinct black‐crowned head. Photo credit: Dhritiman Mukherjee.

Traditional approaches for tracking animals were to follow animals using antennas and record a few dozen locations with high chances of error, revealing only the most general patterns of animal space use (Kays et al. 2015). Enquiring fine‐scale information about animal movement has become possible with the advancement of biologging technologies. Recent improvements have strengthened our ability to study various animal movements with high precision (Hebblewhite and Haydon 2010; Kays et al. 2015; Habib et al. 2021). Consequently, these advancements enable the documentation of movement characteristics of rare species that are typically hard to follow due to their undetectability and low numbers (Cooper and Marra 2020). The critically endangered great Indian bustard ( *Ardeotis nigriceps* ) was believed to roam opportunistically over the landscape, following areas of increased productivity and concentrations of food availability (Ali and Ripley 1969). It inhabits mainly arid and semi‐arid regions of the Indian subcontinent (Figure 1). Among the different biogeographic regions, arid and semi‐arid are categorized by high temperature, low rainfall and less water availability, with shrublands and vast grasslands (Mortimore 1989). The special characteristics of these drylands support a variety of unique biodiversity in these regions (Khan et al. 2020). Although it has a rich biodiversity of flora and fauna, these areas are categorized as ‘wastelands’ and used for various infrastructure developments and incentive lands (Dutta et al. 2010). GIBs were once found in large flocks in these grasslands throughout India and southern Pakistan; their number decreased drastically by the end of the 19th century (Islam and Rahmani 2002). Their population has decreased by 80% in the last 50 years due to hunting, habitat fragmentation and degradation (Khan et al. 2019). The recent decline in their number has been recorded from all present distributional ranges (Dutta et al. 2010). The current populations of Maharashtra, Karnataka, Telangana and Andhra Pradesh are in danger of becoming extinct locally (Khan et al. 2019).

GIB is typically observed in grasslands during the mating season (April–July) because it requires undisturbed, open grasslands for courtship displays and mating (Habib et al. 2016). Outside the breeding season, GIBs are often seen foraging in croplands and travelling long distances (Bhushan and Rahmani 1992). This highlights the need for a landscape‐level conservation approach, as GIBs rely on large, interconnected areas for various activities due to their unique behaviour and habitat needs. However, conserving GIB habitats is challenging because these areas are often located in regions experiencing rapid industrialization, extensive agriculture and growing demands for clean and green energy. Traditional land use practices, such as managed grazing and dry farming, have been beneficial for GIB conservation in the past, but these practices are increasingly at risk.

In this study, we aimed to understand GIB ecology by examining their movement patterns, habitat use and key areas of activity. Specifically, we analyzed daily movement patterns, seasonal variations in movement and monthly overlap in habitat use of our single‐tagged individual. We identified critical areas used by tagged GIB during the study period and investigated the habitat characteristics and human‐related factors influencing their movements. This study provides the first detailed account of GIB's movement behaviour. We hope our findings will contribute to understanding GIB movements and habitat use in human‐dominated landscapes, offering valuable insights for conservation and management efforts.

## 2 Materials and Methods

### 2.1 Study Area

**FIGURE 2:**
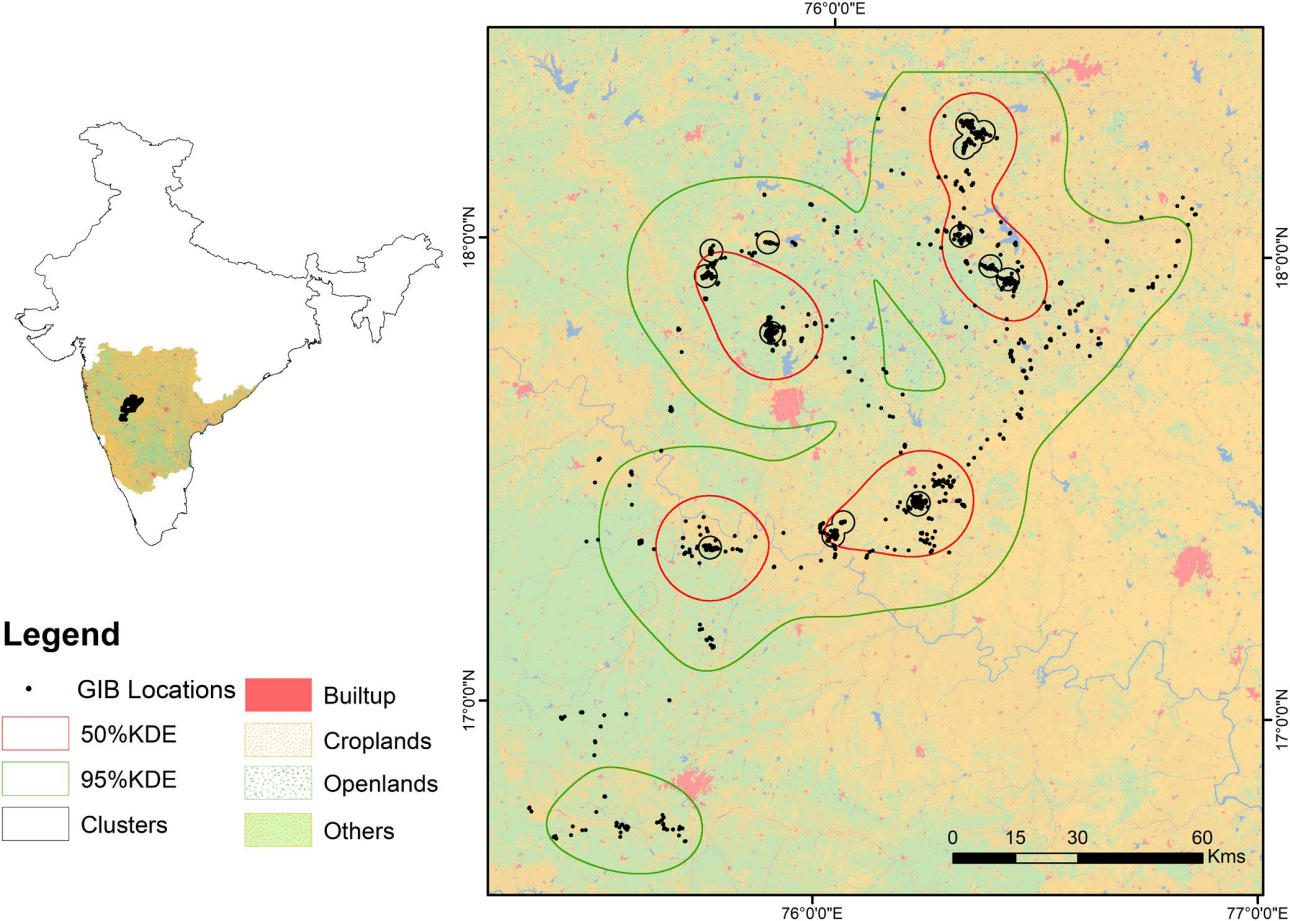
Distribution pattern of tagged GIB, 50 (red) and 95% (green) utilization distribution map using kernel density estimates (KDE) and identified movement clusters (black) in Maharashtra and Karnataka for the tagged individual.

The bird was captured from Nannaj Bustard Sanctuary (17°49′36.2″ N 75°52′10.9″ E), situated north of Solapur, Maharashtra. During the tracking period, the bird roamed in the semi‐arid landscapes of the Solapur and Osmanabad districts of Maharashtra and the Bijapur districts of Karnataka. The region lies in Biogeographic Zone 6 (Deccan Peninsula). The soil type is predominantly laterite, and the black volcanic and basaltic types have average temperatures between 15°C in winter and 44°C in summer. The area receives significantly less rainfall, and the average rainfall in this region is between 600 and 635 mm. The vegetation of the area is classified as Type 6; Tropical Thorn Forest, Subgroup 6A/C1; Southern Tropical Thorn Forest (Champion and Seth 1968). Acacia and Zizyphus dominate the region. The study area has a mosaic of agricultural lands dominated by Kharif crops and patches of grassland with bare intervening grounds covered with exposed rocks and stones (Figure 2). In the study area, the major crops are *Sorghum bicolor* , *Pennisetum glaucum* , *Zea mays* , *Cajanus cajan* , *Gossypium* sp. and *Arachis hypogaea*.

### 2.2 Capturing and Tagging the Individual

Capturing a GIB is a highly challenging and delicate task due to its endangered status and the open habitats it occupies. In April 2015, a sub‐adult male Great Indian Bustard was captured in Nannaj Bustard Sanctuary using noose traps, a method specifically selected for its minimal disturbance to the bird and surrounding environment.

The process began with extensive preparatory work, including observing the bird's movement patterns, feeding behaviour and drinking schedule. The field team spent considerable time identifying frequently visited locations, which helped in strategically placing the noose traps. The placement ensured that the bird would naturally walk into the trap while foraging or moving near the waterhole without detecting any disturbance.

To minimize stress and ensure the safety of the birds, the team closely monitored the trap sites from a hide. Once the bird was trapped, we quickly approached it to minimize distress. After capture, the sub‐adult male was carefully examined for overall health, physical condition and any signs of injury. A 70‐g Solar Argos/GPS platform transmitting terminal (PTT) manufactured by Microwave Telemetry Inc. was fitted using a harness. The PTT recorded seven GPS locations daily in peak GIB activity hours from 05:30 to 19:30 h (5:30, 07:30, 09:30, 11:30, 13:30, 17:30 and 19:30) and multiple Argos locations on alternate days. The bird was tracked till April 2016, and we used GPS and class‐3 Argos locations to evaluate movement parameters, utilization distribution and identification of movement clusters to understand species ecology.

### 2.3 Data Analyses

From April 2015 to June 2016, the tagged individual was monitored for 410 days. A total of 3345 locations were collected, comprising 2861 GPS and 484 Class‐3 Argos locations, which were used for the analysis.

### 2.4 Movement Analysis

To investigate the overall movement behaviour of the individual, we applied the Lévy flight model to the full dataset of 3345 GPS locations. The analysis focused on fitting a power‐law distribution to the step lengths (i.e., the straight‐line distances between successive GPS fixes). This analysis was also conducted on seasonally segmented data (summer, March–June; monsoon, July–September; post‐monsoon, October–November and winter, December–February) to examine potential differences in movement strategies across seasons. We used R packages, fitdistrplus (Delignette‐Muller and Dutang 2015) and poweRlaw (Gillespie 2015) to estimate the scaling exponent (*μ*), which characterizes whether the movement followed a Lévy pattern, a strategy often associated with optimal search behaviour (Barbosa et al. 2018). The *μ* value falls within the theoretical Lévy range (1 < *μ* < 3), indicating a pattern characterized by numerous short movements interspersed with occasional long‐distance displacements. Such a pattern is considered optimal for searching in environments with sparsely and unpredictably distributed resources.

We then analysed the distribution of step lengths and directionality to assess movement dynamics at a finer scale. These values formed the basis for our daily movement calculations. To estimate daily movement, we summed the step lengths recorded across each day, providing a measure of the total distance travelled per day. This approach captured the overall displacement on a daily scale and was implemented using the amt package in R (Signer et al. 2019) and directionality was assessed using the geosphere package in R (Karney 2013). In addition, we also examined seasonal variations in daily movement for four seasons. For each season, we analysed both the scaling exponent from the Lévy model and daily movement patterns, enabling a comparison of how movement strategies may vary with changing environmental conditions.

### 2.5 Utilization Distribution and Monthly Overlap

To evaluate the spatial use patterns of the GIB, we estimated utilization distributions using kernel density estimation (KDE). KDE is a widely used method to represent the probability of an animal's occurrence across space based on location data. We generated KDE surfaces using the ArcMET tool in ArcGIS 10.8 (Wall 2014). Utilization distributions were calculated at two levels: the 50% contour, representing the core area of use, and the 95% contour, representing the overall range. These contours allowed us to identify areas most intensively used by the individual and to assess broader patterns of space use across the landscape.

The monthly overlap between two consecutive months from April 2015 to June 2016 was estimated using 95% KDE.

To understand GIB's movement patterns, we used the amt package to examine the overlap between different utilization distributions, with the result ranging from 0 (*no overlap*) to 1 (*complete overlap*).

To evaluate the spatial use patterns, we estimated utilization distributions (UDs) using the KDE method. KDE is a widely used non‐parametric approach that allows for the creation of a continuous surface indicating the probability of an animal's presence across a landscape based on GPS location data. We generated KDE surfaces using the ArcMET tool in ArcGIS 10.8 (Wall 2014). UDs were computed at two isopleths: 50% KDE contour, representing the core area of use, and 95% KDE contour, representing the broader range. These contours enabled us to delineate both the most intensively used areas and the overall extent of spatial use by the tracked individual.

To investigate temporal changes in space use, we calculated monthly 95% KDEs from April 2015 to June 2016 and quantified spatial overlap between successive months. This was done to assess consistency or shifts in space use patterns over time. We quantified the degree of spatial overlap using the amt package in R (Signer et al. 2019), which computes the overlap between utilization distributions on a scale from 0 (*no overlap*) to 1 (*complete overlap*). This allowed us to capture the dynamic nature of the GIB's movement and range fidelity across different months.

### 2.6 Movement Clusters

Movement clusters were used to pinpoint key areas where the GIB spent significant time on activities like foraging. By identifying these areas, we could gain insights into the species' habitat preferences and movement behaviour. Understanding these clusters helps highlight critical regions that support the GIB's survival, making them potential targets for focused conservation efforts. Protecting such areas can ensure the availability of essential resources and mitigate threats from habitat loss or human activities, ultimately contributing to the conservation of the species in its natural landscape.

To identify movement clusters during the tracking period, we used the GPSeqClus package (Clapp et al. 2021). This package applies a clustering algorithm designed to group locations based on time‐series spatial data. Specifically, the algorithm works by aggregating locations that fall within a defined spatial and temporal threshold. For the analysis, we set the spatial search radius to 2702 m, which corresponds to the average daily movement of the tracked individuals. The temporal window was set to 4 days, meaning locations recorded within this time frame were considered for clustering. Additionally, we required a minimum of 28 locations to form a cluster, ensuring the identified clusters were meaningful and robust. We selected a 4‐day temporal window and a minimum of 28 locations per cluster based on both biological reasoning and the characteristics of the GPS dataset. The 4‐day window was chosen to balance between capturing short‐term spatial use (e.g., resting or foraging areas) and allowing enough time for the animal to potentially show site fidelity or repeated use of a specific area. Shorter windows (e.g., 1–2 days) risked identifying transient pauses in movement as clusters, which may not represent true areas of importance. A 4‐day span provides a more ecologically meaningful period to detect behaviourally relevant site use without being overly restrictive. The requirement of at least 28 locations per cluster was based on the GPS data recording frequency, which provided approximately 7 locations per day. This threshold ensures that clusters represent at least four consecutive days of consistent space use. This reduces noise and increases confidence that identified clusters reflect biologically meaningful behaviour. This approach allowed us to systematically identify and analyze areas of concentrated activity, providing insights into movement patterns and habitat use.

We assessed the habitat configuration of these clusters by evaluating land cover percentages, mean NDVI, distance from powerlines and road density. NDVI data were obtained from Google Earth Engine (GEE) between January 2015 and July 2016, with a spatial resolution of 30 m and a temporal resolution of 32 days (Gorelick et al. 2017). NDVI values, which range from 0 to 1, were extracted for each cluster's start date.

We also extracted road network and powerline data from Open Street Maps (https://www.openstreetmap.org/) to evaluate the proximity of each movement cluster to these features. The cluster's habitat configuration was analyzed based on land use proportions, road density, mean NDVI and distance from the nearest powerline. All calculations and statistical analyses were performed using R 4.1.3 (R Core Team 2024), ArcGIS (10.8) and the Google Earth Engine platform.

## 3 Results

**FIGURE 3:**
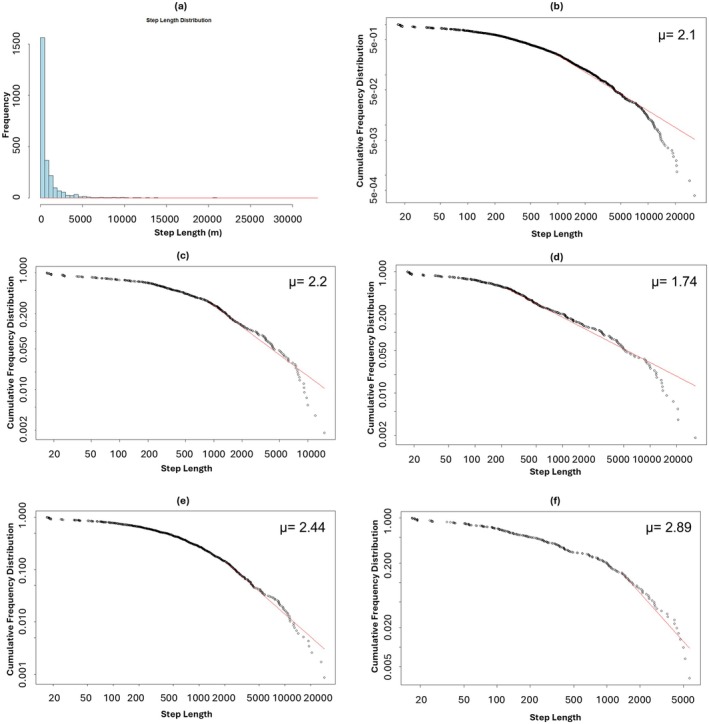
Step length distribution and power‐law model fitting of GPS‐based movement data (a) Step length distribution of the overall movement data (*n* = 3345 GPS fixes), (b) fitted power‐law model for the overall dataset, (c) monsoon season, (d) winter season, (e) summer season, (f) post‐monsoon season. Each panel (b–f) shows the fitted power‐law curve along with the estimated scaling exponent (*μ*), characterising the movement type. Values of *μ* between 1 and 3 are indicative of Lévy flight movement, while values close to 3 suggest a mix of Lévy flight and Brownian‐like random movement.

We recorded a total of 3345 GPS fixes, and the result showed that the individual moved 0.33 km/h (range: 0–10.4 km/h). The log–log plot of the empirical data versus the fitted distribution (Figure 3) supports this finding, showing a linear trend in the tail of the distribution, which is a hallmark of power‐law behaviour. The individual showed a Lévy flight movement pattern with a scaling exponent (*μ*) value of 2.10. The data were divided into four seasons: monsoon, winter, summer and post‐monsoon. During the monsoon, winter and summer seasons, the individual exhibited a Lévy flight movement pattern, with μ values of 2.20, 1.74 and 2.44, respectively. However, in the post‐monsoon season, the estimated μ value was 2.89, which is closer to Brownian‐like movement, indicating a more random and less scale‐free movement pattern during this period.

The sub‐adult GIB travelled 2208.66 km in total during the tracking period from April 2015 to June 2016, and its average daily movement was calculated to be 5.39 km ±5.58 SD (range: 0.17–38.99). The maximum distance travelled in a day was calculated to be 38.9 km, traversed from the Nannaj Bustard Sanctuary (17^0^48′39.6″ N‐75^0^52′54.59″ E) to the Akkalkot area (17^0^27′6.59″ N‐76^0^13′16.79″ E) of Solapur District.

**FIGURE 4:**
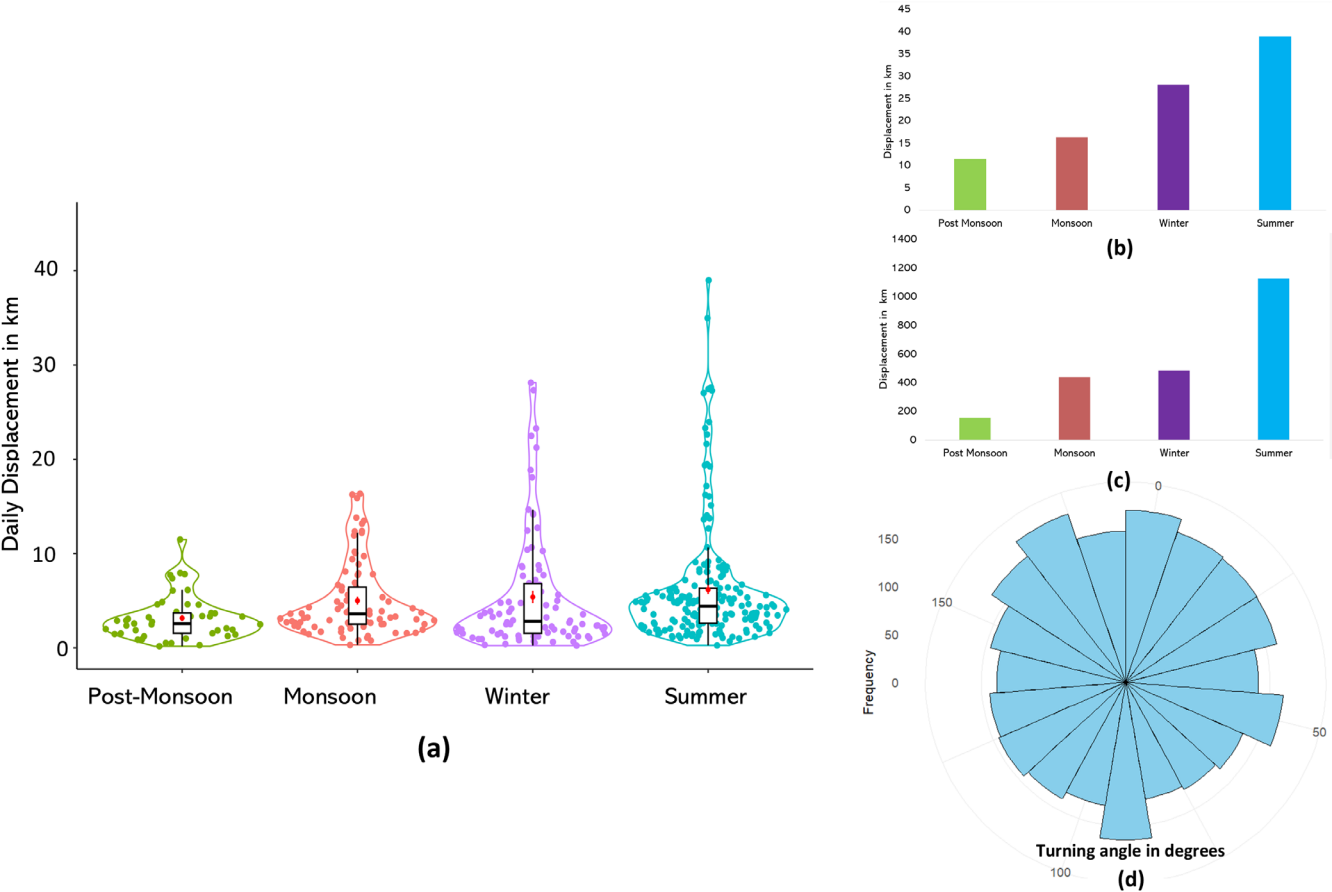
Movement pattern of GIB in different seasons: (a) daily movement in different seasons [the black dots denoting the average daily movement], (b) maximum displacement in 1 day in different seasons, (c) sum of total displacement in different seasons, (d) directionality histogram illustrating the frequency distribution of turning angles in the movement path.

The daily displacement was varied in different seasons, i.e., in summer, winter, monsoon and post‐monsoon. The highest daily displacement was found in summer (6.17 km/day; range: 0.26–38.99), followed by winter (5.39 km/day; range: 0.24–28.12) and lowest was in post‐monsoon 3.15 km/day (range: 0.17–11.49) (Figure 4). The total movement of GIB was found to be maximum in the summer (1128.68 km), followed by the winter (485.14 km) and lowest in the post‐monsoon (154.59 km) (Figure 4). The individual showed no directional persistence over the tracking period (Figure 4).

**FIGURE 5:**
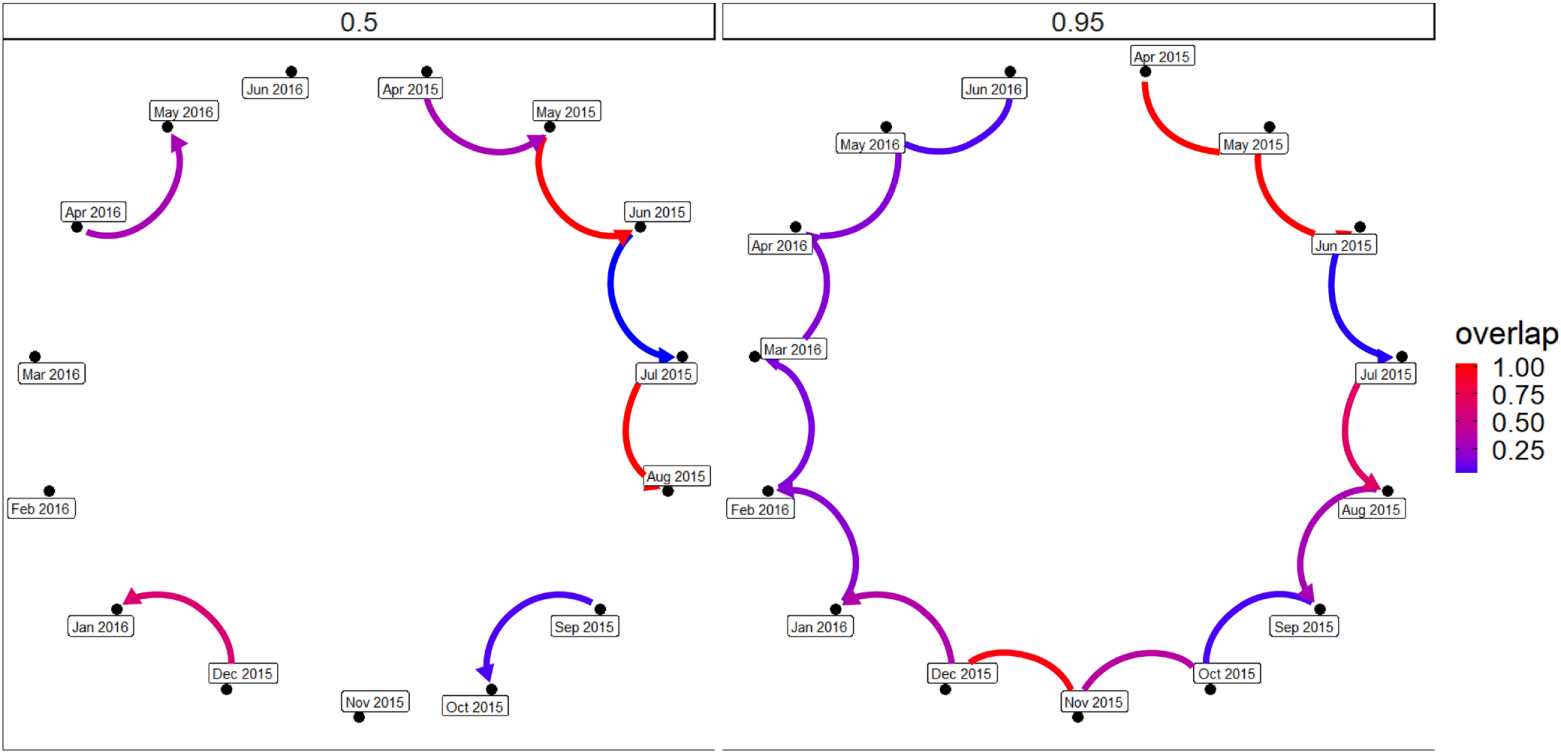
Monthly utilization distribution overlap of GIB using 95% Kernel Density Estimator (KDE). The dark red shows a 100% overlap, whereas dark blue shows a < 25% overlap in the monthly utilization distribution. No connections between the months show non‐overlap.

We report the utilization distribution of GIB using KDE for 50% and 95% contours. The total core area (50% KDE) of 2633.07 km^2^ was divided into four clusters (mean ± SD = 688.89 km^2^ ±173.224). The 95% KDE or utilization distribution was estimated to be 12080.78 km^2^ (Figure 2). The monthly overlap in the utilization distribution showed that the monthly overlap exceeded 50% on only four occasions. The overlap for April and May in 2015 and 2016 was 52% and 55%, respectively. For July–August in 2015 and December–January in 2016, the overlap was 63% and 65%, respectively, and the overlap was under 50% throughout the rest (Figure 5).

We identified 10 movement clusters formed during the tracking period in different regions of Maharashtra and Karnataka (Figure 2). The identified movement clusters were dominated by open lands (fallow lands, degraded lands and grasslands), followed by Kharif and mixed crops (43.29%). The mean distance from the high‐tension powerlines was found to be 2919.38 m ±3011.05SD. The mean road density for all the movement clusters was found to be 0.38 km/km^2^ ±0.24SD. The mean NDVI value for these clusters was 0.31, ranging from 0.16 to 0.43. The details for each cluster are provided in Table 1.

**TABLE 1:** Details of 10 movement clusters identified based on movement data of GIB in the states of Maharashtra and Karnataka, India. All the parameters were calculated in the identified cluster with a diameter of 5 km based on the average daily movement of GIB.

Clusters	Lat–Long	Area	Builtup	Crop	Open	Others	Water	Nearest powerline (m)	Road density (km/km^2^)	NDVI mean
1	17.8132, 75.87388	22.94	2.06	22.22	75.40	0.21	0.11	840.34	0.58	0.23
2	18.00682, 75.86083	22.94	1.16	48.04	49.55	0.00	1.25	2891.83	0.09	0.21
3	17.45103, 76.21547	25.42	0.61	62.95	33.56	0.00	2.87	0.00	0.71	0.25
4	17.93501, 76.40594	45.80	1.74	23.95	71.67	0.03	2.61	10318.24	0.40	0.43
5	18.02985, 76.29699	22.94	0.03	31.46	65.61	0.60	2.31	2309.19	0.31	0.36
6	18.25485, 76.32934	71.81	0.89	44.05	49.77	1.93	3.37	502.66	0.14	0.16
7	17.9312, 75.72263	22.94	1.35	37.07	57.32	2.10	2.17	5277.44	0.23	0.42
8	17.98588, 75.73365	22.94	0.92	41.19	55.96	0.08	1.85	1872.52	0.25	0.39
9	17.37725, 76.02758	42.48	0.05	77.22	22.73	0.00	0.00	3270.37	0.25	0.39
10	17.34404, 75.74915	22.94	0.46	44.77	53.33	0.13	1.32	1911.17	0.80	0.30
		Mean	0.93	43.29	53.49	0.51	1.79	2919.38	0.38	0.31
		±SD	0.67	16.88	16.16	0.81	1.12	3011.05	0.24	0.10

## 4 Discussion

The present study describes the GIB movement patterns, utilization distribution and identification of critical conservation areas in the semi‐arid landscapes of Maharashtra and Karnataka states, India. To our knowledge, this study is the first to document the GIB movement. We documented the GIB movement in the Deccan Landscape, which varied noticeably and had particular cropping patterns. We conducted a study on a single individual to understand the ecology of birds. Logistical constraints and low GIB in the area limited the number of transmitters deployed. Nevertheless, the study presented detailed information on one individual's movement pattern, which is unprecedented for a critically endangered species. The study's findings could help plan better conservation efforts to save the bird from extinction.

In this study, we tracked the movement patterns of an individual using GPS data, recording a total of 3345 fixes. The average hourly movement rate was 0.33 km/h, with values ranging from 0 to 10.4 km/h. These findings indicate that the individual exhibited relatively slow, consistent movement, with some periods of faster travel, possibly influenced by factors such as landscape features or behavioural responses (Edwards 2011).

The GIB followed a Lévy flight movement pattern across the entire tracking period, with a scaling exponent (*μ*) of 2.10, which is consistent with previous studies that have observed Lévy flights in animal movement behaviour. Lévy flight patterns are characterized by long, irregular movements interspersed with short, local movements, which are thought to be an optimal strategy for foraging in complex environments (Van Houtan et al. 2007). This type of movement is typically found in animals searching for sparse and unpredictable resources, suggesting that the individual may have been optimizing their search behaviour (Ríos‐Uzeda et al. 2019).

The seasonal variation in the *μ* values derived from the Lévy flight analysis provides valuable insights into the movement strategies of the GIB across different times of the year. These values, 2.89 (post‐monsoon), 2.20 (monsoon), 1.74 (winter) and 2.44 (summer), reflect shifts in the bird's ranging behaviour likely influenced by changing ecological conditions, resource distribution and behavioural needs.

During the post‐monsoon season, the high *μ* value of 2.89 indicates a Brownian‐like movement pattern, suggesting that the bird's movement was highly localized, possibly due to the availability of food and water resources post‐rainfall, reducing the need for long‐distance exploration. The GIB might have been utilizing a well‐known, resource‐rich area during this time, resulting in more intensive and area‐restricted foraging. In contrast, the winter season showed the lowest *μ* value (1.74), clearly reflecting a Lévy flight pattern. This suggests a more exploratory movement behaviour, potentially driven by reduced availability of resources or the need to access widely distributed habitats (Brigatti et al. 2024). Such a strategy enables the bird to increase its chances of encountering scarce resources.

The monsoon season showed an intermediate μ of 2.20, leaning closer to a Lévy‐type strategy. The landscape during the monsoon can be patchy due to temporary water bodies and rapid vegetation growth, which might encourage broader but still partially localized movements. Finally, the summer *μ* value of 2.44 suggests a mixed or moderately diffusive movement strategy. During this period, GIBs might balance between exploiting known refuges and searching for new areas as resources become increasingly limited due to dry conditions.

The circular histogram of turning angles showed a uniform distribution across all directions, with no dominant peaks. This pattern indicated that the movement of the GIB lacks directional persistence, meaning the individual frequently changes direction rather than maintaining a consistent trajectory. Such movement behaviour aligns with the nomadic lifestyle of the GIB. As a nomadic species, the GIB does not follow fixed routes or display site fidelity to specific foraging patches. Instead, it utilizes an area for a limited period and then moves randomly to a new location in search of better resources. This erratic and non‐linear movement results in a wide range of turning angles over time, reflected in the circular histogram (Figure 4).

The lack of directional persistence may be influenced by several ecological factors such as patchy resource distribution and existing disturbances. In the semi‐arid grasslands, resources like food and water are often scattered, prompting the birds to frequently change direction in search of new foraging patches. Disturbance and habitat fragmentation due to human activity and land use changes may interrupt straight‐line movements, causing more frequent turning.

During the tracking period of 410 days, the GIB traversed 2208.66 km and crossed major powerlines on 67 occasions. However, the area is crisscrossed by numerous minor powerline networks, which could potentially deter GIB flying movement. As a result, they are one of the significant threats to GIB conservation since they can collide with power lines (Uddin et al. 2021). The study suggested that the movement strategies of the GIB may be complex and could vary among seasons. The GIB showed the highest movement in summer, 6.17 km/day, and the lowest in pre‐monsoon, 3.15 km/day. This may be because there was a shortage of food and cover throughout the summer, but during the pre‐monsoon, agricultural fields were active, and there was plenty of food in the form of insects and seeds, and it also provided good cover (BirdLife International 2025) as previously reported for other bird species (Katuwal et al. 2016). The inconstant movement strategies are common for long‐ranging species, particularly those that follow particular environmental conditions (Nandintsetseg et al. 2019). These differential movements adapt to the specific notions of local environmental conditions (Ziembicki and Woinarski 2009; Ziembicki 2009). The movement pattern revealed that GIB utilizes a particular area for a certain period and then moves to a new area. This pattern was observed throughout the tracking period.

Nomadic movement, driven by environmental variability and habitat availability, is characterized by wide‐ranging and irregular patterns (Teitelbaum and Mueller 2019). Despite its ecological significance, this movement type is poorly understood (Mueller and Fagan 2008), likely due to the difficulty of tracking such species. Long‐ranging animals are especially vulnerable to anthropogenic pressures, so studying their movement for effective conservation is crucial. The nomadic movement behaviour often provides large and non‐static utilization distribution estimates (Roshier and Reid 2003). Our study showed that the overall utilization distribution (95% KDE) during the tracking period was 12080.78 km^2^, whereas the core area (50% KDE) was 2633.07 km^2^. There are currently no comprehensive studies of the Great Indian Bustard's biology to compare the results of this study. The study on utilization distribution (95% KDE) of the Houbara bustard ( *Chlamydotis undulata* ) in China was 274 km^2^ (Judas et al. 2006). In Taiwan, Short‐eared owls were found to have an average core area of 158.9 ha and a home range of 1268.7 ha. These large home ranges reflect their nomadic lifestyle and the necessity to cover extensive areas to meet their ecological needs (Tseng et al. 2017).

Other studies on Grey teal ( *Anas gracilis* ) in the arid environment also reported large utilization distribution (Roshier et al. 2006). These studies highlight the significant variability in home range sizes among nomadic bird species, influenced by factors such as resource distribution, environmental conditions and species‐specific behaviours. We found that tagged individual GIB also has an extensive estimated utilization distribution using KDE. This large area is 10 times larger than the designated protected area for GIB conservation in the landscape (The Great Indian Bustard Sanctuary, Nannaj, Maharashtra). The GIB used protected areas sporadically throughout the tracking period, indicating that PAs are ineffective conservation measures for the species. The individual utilization distribution of over 12,000 km^2^ indicates that conservation efforts must extend beyond the current protected area boundaries. Hence, landscape‐level management is essential for effectively conserving the species in this region.

Nomadic species move between spatially separated sites rather than remaining in a static home range (Roshier and Reid 2003). The low monthly overlap in the utilization distribution suggests significant variability in space use, likely influenced by seasonal changes in environmental conditions, resource availability and habitat preferences. Seasonal shifts in habitat conditions, such as food and water availability, are known to drive changes in spatial overlap. Studies on nomadic species indicate higher overlap during periods of stable resources and lower overlap when resources are patchy or ephemeral (Teitelbaum and Mueller 2019). For instance, migratory and nomadic birds often congregate during breeding seasons or other critical life‐history events, which may explain the observed overlaps during April–May and July–August. In contrast, reduced overlap during other months likely reflects foraging movements to exploit widely distributed or temporary resources (Mueller and Fagan 2008).

Anthropogenic pressures and climatic variability may further reduce spatial overlap. Fragmented landscapes and habitat loss due to human activities force species to adjust their spatial use, often leading to decreased overlap in utilization patterns (Tucker et al. 2018). For example, the GIB also shows shifts in utilization distribution due to resource variability and anthropogenic disturbances (Dutta et al. 2010). Similarly, wetland‐dependent species like the Grey Teal ( *Anas gracilis* ) exhibit significant seasonal variations in space use driven by fluctuating water levels (Roshier et al. 2006).

Over the last 100 years, India has experienced a significant loss of grasslands and shrublands due to various anthropogenic activities (Tian et al. 2014). In addition, the increasing network of canals in semi‐arid landscapes encourages farmers to shift to cash crops, resulting in the rapid decline in traditional cropping patterns (pulses and food crops) in the landscape (Nalawade et al. 2010; Seethalakshmi 2010). The change in the cropping pattern, conversion of undisturbed grasslands and shrublands into agricultural lands, overgrazing of pasture and human disturbance could lead to GIB extermination from the landscape. Due to the poor frontal vision of GIB, the powerlines are a major threat to the population. We also calculated the distance of powerlines from the edge of each cluster boundary and found only two movement clusters near powerlines, suggesting the GIB's avoidance of major powerlines. The average road density was found to be 0.72 km/km^2^ ±0.49SD.

The previous studies on bustards highlighted that they follow rainfall patterns. To understand this, we also evaluated NDVI in each cluster. The NDVI for each cluster was evaluated from the nearest available data date when the bustards used the cluster. We have found that GIB prefers a close range of NDVI (0.31 ± 0.1SD) throughout the tracking period. This suggests that GIB follows rainfall to track Kharif and rabi crops in the landscape for food and cover.

This study provides a comprehensive investigation into the movement patterns, utilization distribution and identification of critical foraging areas for the GIB. It represents the first detailed analysis of GIB's movement parameters using satellite telemetry, offering new insights into the species' space use and behaviour. The study highlights irregular, nomadic movement patterns driven by the need to follow suitable habitats and climate conditions. It also identifies critical movement clusters where the GIB spent significant time foraging, demonstrating the species' reliance on a variety of landscapes within its range. These findings suggest that GIB movements are not confined to fixed locations but are highly influenced by environmental factors, with the region consisting of an interconnected mosaic of different land use types.

However, it is important to note that this study is based on data from a single individual, which limits the ability to extrapolate the results to the broader population of GIBs. Tagging and tracking additional individuals would provide a more robust dataset, enabling a deeper understanding of the species' movement ecology across different habitats and seasons. While this study offers valuable baseline data, it would benefit from further research to capture the variability in movement patterns across individuals and populations. This would enhance our understanding of GIB ecology and contribute to more effective management strategies for the species.

## Author Contributions

**Shaheer Khan:** conceptualization (equal), data curation (lead), formal analysis (lead), funding acquisition (equal), investigation (supporting), methodology (lead), project administration (equal), resources (equal), software (equal), supervision (equal), validation (equal), visualization (equal), writing – original draft (lead), writing – review and editing (lead). **Vaijayanti Vijayaraghavan:** data curation (equal), methodology (equal), visualization (equal), writing – review and editing (equal). **Gautam Talukdar:** conceptualization (supporting), data curation (supporting), formal analysis (supporting), funding acquisition (equal), investigation (equal), methodology (supporting), project administration (equal), supervision (equal), validation (equal), writing – review and editing (equal). **R. Suresh Kumar:** conceptualization (supporting), data curation (supporting), formal analysis (supporting), funding acquisition (equal), investigation (equal), methodology (supporting), project administration (equal), supervision (equal), validation (equal), writing – review and editing (equal). **Bilal Habib:** conceptualization (lead), data curation (lead), formal analysis (supporting), funding acquisition (lead), investigation (lead), methodology (equal), project administration (lead), resources (lead), software (lead), supervision (lead), validation (lead), visualization (equal), writing – original draft (equal), writing – review and editing (equal).

## Ethics Statement

The bird was captured following standard and approved protocols approved by the Wildlife Institute of India, Animal Ethics Committee, after permission from the Ministry of Environment, Forests and Climate Change, Government of India and Maharashtra Forest Department. The species‐specific permit detail to capture and tag the birds is as follows: SPP‐01 (2015) dated 08.04.2015.

## Conflicts of Interest

The authors declare no conflicts of interest.

## Supporting information


Appendix S1.

## Data Availability

The data sets generated and analyzed in the present study, along with the analysis codes, are provided as Supporting Information.
