# Supplementary material for: Not All Those Who Wander Are Lost: Insights Into Movement Pattern of the Great Indian Bustard in the Deccan Landscape of India
Source: Ecol Evol. 2025 Jul 6;15(7):e71742. doi: 10.1002/ece3.71742 (PMC12230192; doi:10.1002/ece3.71742)
Supplement: Supplementary file 1 — Appendix S1. [file ECE3-15-e71742-s001.docx]

**Daily Movement of Great Indian Bustard in the Deccan Landscape of Maharashtra**

| **S.no.** | **StartDate** | **EndDate** | **FiltSegCount** | **Daily displacement in km** | **Daily displacement in meters** | **Standard Deviation** |
| --- | --- | --- | --- | --- | --- | --- |
| 1 | 4/16/2015 | 4/17/2015 | 8 | 1.03 | 1032.9 | 14.8 |
| 2 | 4/17/2015 | 4/18/2015 | 5 | 1.47 | 1466.2 | 22.3 |
| 3 | 4/18/2015 | 4/19/2015 | 5 | 0.26 | 257.8 | 6.9 |
| 4 | 4/19/2015 | 4/20/2015 | 5 | 4.75 | 4746.2 | 29.3 |
| 5 | 4/20/2015 | 4/21/2015 | 5 | 3.05 | 3051.6 | 23.4 |
| 6 | 4/21/2015 | 4/22/2015 | 5 | 5.52 | 5516.8 | 37.5 |
| 7 | 4/22/2015 | 4/23/2015 | 7 | 3.61 | 3608.8 | 20.6 |
| 8 | 4/23/2015 | 4/24/2015 | 5 | 5.66 | 5662.3 | 27.8 |
| 9 | 4/24/2015 | 4/25/2015 | 8 | 2.39 | 2390.0 | 16.8 |
| 10 | 4/25/2015 | 4/26/2015 | 5 | 4.40 | 4404.4 | 23.0 |
| 11 | 4/26/2015 | 4/27/2015 | 7 | 5.06 | 5063.7 | 28.6 |
| 12 | 4/27/2015 | 4/28/2015 | 5 | 5.22 | 5223.8 | 37.4 |
| 13 | 4/28/2015 | 4/29/2015 | 5 | 5.99 | 5991.0 | 27.9 |
| 14 | 4/29/2015 | 4/30/2015 | 5 | 5.34 | 5337.7 | 35.3 |
| 15 | 4/30/2015 | 5/1/2015 | 6 | 4.64 | 4635.4 | 34.2 |
| 16 | 5/1/2015 | 5/2/2015 | 5 | 9.10 | 9104.4 | 46.6 |
| 17 | 5/2/2015 | 5/3/2015 | 8 | 7.54 | 7540.0 | 32.9 |
| 18 | 5/3/2015 | 5/4/2015 | 5 | 3.77 | 3765.4 | 23.2 |
| 19 | 5/4/2015 | 5/5/2015 | 6 | 3.80 | 3795.2 | 20.9 |
| 20 | 5/5/2015 | 5/6/2015 | 5 | 2.20 | 2197.8 | 22.6 |
| 21 | 5/6/2015 | 5/7/2015 | 8 | 3.58 | 3576.4 | 17.5 |
| 22 | 5/7/2015 | 5/8/2015 | 5 | 3.48 | 3479.1 | 27.3 |
| 23 | 5/8/2015 | 5/9/2015 | 6 | 3.03 | 3035.0 | 28.4 |
| 24 | 5/9/2015 | 5/10/2015 | 5 | 2.04 | 2038.8 | 21.0 |
| 25 | 5/10/2015 | 5/11/2015 | 9 | 14.05 | 14048.2 | 34.0 |
| 26 | 5/11/2015 | 5/12/2015 | 5 | 1.23 | 1233.0 | 15.1 |
| 27 | 5/12/2015 | 5/13/2015 | 6 | 6.33 | 6328.3 | 38.3 |
| 28 | 5/13/2015 | 5/14/2015 | 5 | 2.65 | 2647.2 | 20.6 |
| 29 | 5/14/2015 | 5/15/2015 | 7 | 3.36 | 3362.4 | 20.4 |
| 30 | 5/15/2015 | 5/16/2015 | 5 | 5.44 | 5444.3 | 27.0 |
| 31 | 5/16/2015 | 5/17/2015 | 6 | 3.55 | 3550.1 | 25.2 |
| 32 | 5/17/2015 | 5/18/2015 | 5 | 5.13 | 5128.7 | 25.6 |
| 33 | 5/18/2015 | 5/19/2015 | 7 | 3.47 | 3472.9 | 22.6 |
| 34 | 5/19/2015 | 5/20/2015 | 5 | 4.30 | 4303.8 | 29.2 |
| 35 | 5/20/2015 | 5/21/2015 | 8 | 4.74 | 4738.6 | 22.2 |
| 36 | 5/21/2015 | 5/22/2015 | 5 | 4.79 | 4792.7 | 25.2 |
| 37 | 5/22/2015 | 5/23/2015 | 5 | 4.19 | 4185.8 | 24.1 |
| 38 | 5/23/2015 | 5/24/2015 | 5 | 4.81 | 4815.0 | 28.1 |
| 39 | 5/24/2015 | 5/25/2015 | 5 | 5.01 | 5014.2 | 24.8 |
| 40 | 5/25/2015 | 5/26/2015 | 5 | 4.70 | 4696.7 | 34.2 |
| 41 | 5/26/2015 | 5/27/2015 | 8 | 7.19 | 7192.7 | 28.3 |
| 42 | 5/27/2015 | 5/28/2015 | 5 | 4.98 | 4980.9 | 25.3 |
| 43 | 5/28/2015 | 5/29/2015 | 8 | 5.53 | 5534.5 | 19.6 |
| 44 | 5/29/2015 | 5/30/2015 | 5 | 2.97 | 2973.1 | 21.1 |
| 45 | 5/30/2015 | 5/31/2015 | 6 | 8.23 | 8229.2 | 42.0 |
| 46 | 5/31/2015 | 6/1/2015 | 5 | 8.61 | 8605.2 | 53.2 |
| 47 | 6/1/2015 | 6/2/2015 | 8 | 8.56 | 8560.9 | 34.5 |
| 48 | 6/2/2015 | 6/3/2015 | 5 | 5.49 | 5493.7 | 29.3 |
| 49 | 6/3/2015 | 6/4/2015 | 7 | 1.44 | 1439.6 | 12.5 |
| 50 | 6/4/2015 | 6/5/2015 | 5 | 1.01 | 1006.7 | 17.4 |
| 51 | 6/5/2015 | 6/6/2015 | 6 | 3.72 | 3721.5 | 23.2 |
| 52 | 6/6/2015 | 6/7/2015 | 5 | 3.55 | 3548.2 | 23.4 |
| 53 | 6/7/2015 | 6/8/2015 | 9 | 3.74 | 3743.3 | 18.2 |
| 54 | 6/8/2015 | 6/9/2015 | 5 | 1.29 | 1291.7 | 16.6 |
| 55 | 6/9/2015 | 6/10/2015 | 7 | 2.33 | 2328.6 | 15.4 |
| 56 | 6/10/2015 | 6/11/2015 | 5 | 2.26 | 2256.3 | 16.3 |
| 57 | 6/11/2015 | 6/12/2015 | 8 | 27.46 | 27460.5 | 67.4 |
| 58 | 6/12/2015 | 6/13/2015 | 5 | 27.62 | 27621.5 | 91.1 |
| 59 | 6/13/2015 | 6/14/2015 | 8 | 7.73 | 7730.9 | 32.7 |
| 60 | 6/14/2015 | 6/15/2015 | 5 | 16.22 | 16221.0 | 61.8 |
| 61 | 6/15/2015 | 6/16/2015 | 7 | 15.12 | 15123.6 | 51.5 |
| 62 | 6/16/2015 | 6/17/2015 | 5 | 12.67 | 12665.9 | 45.4 |
| 63 | 6/17/2015 | 6/18/2015 | 7 | 13.74 | 13740.2 | 39.1 |
| 64 | 6/18/2015 | 6/19/2015 | 5 | 27.03 | 27029.9 | 67.6 |
| 65 | 6/19/2015 | 6/20/2015 | 6 | 21.63 | 21626.2 | 68.0 |
| 66 | 6/20/2015 | 6/21/2015 | 5 | 3.23 | 3229.5 | 33.6 |
| 67 | 6/21/2015 | 6/22/2015 | 6 | 10.67 | 10666.5 | 52.8 |
| 68 | 6/22/2015 | 6/23/2015 | 5 | 6.28 | 6277.9 | 34.7 |
| 69 | 6/23/2015 | 6/24/2015 | 7 | 6.29 | 6290.0 | 32.4 |
| 70 | 6/24/2015 | 6/25/2015 | 5 | 2.67 | 2666.4 | 23.7 |
| 71 | 6/25/2015 | 6/26/2015 | 6 | 5.15 | 5146.3 | 24.7 |
| 72 | 6/26/2015 | 6/27/2015 | 5 | 8.20 | 8197.2 | 37.9 |
| 73 | 6/27/2015 | 6/28/2015 | 7 | 1.12 | 1122.3 | 11.1 |
| 74 | 6/28/2015 | 6/29/2015 | 5 | 0.93 | 927.4 | 7.8 |
| 75 | 6/29/2015 | 6/30/2015 | 7 | 38.99 | 38990.8 | 86.4 |
| 76 | 6/30/2015 | 7/1/2015 | 5 | 13.63 | 13631.8 | 75.8 |
| 77 | 7/1/2015 | 7/2/2015 | 7 | 2.92 | 2917.5 | 18.2 |
| 78 | 7/2/2015 | 7/3/2015 | 5 | 2.78 | 2777.8 | 22.0 |
| 79 | 7/3/2015 | 7/4/2015 | 8 | 2.64 | 2643.3 | 13.6 |
| 80 | 7/4/2015 | 7/5/2015 | 5 | 1.07 | 1070.6 | 14.9 |
| 81 | 7/5/2015 | 7/6/2015 | 8 | 4.91 | 4906.7 | 20.7 |
| 82 | 7/6/2015 | 7/7/2015 | 5 | 3.05 | 3054.4 | 21.4 |
| 83 | 7/7/2015 | 7/8/2015 | 7 | 4.36 | 4359.7 | 25.6 |
| 84 | 7/8/2015 | 7/9/2015 | 5 | 4.11 | 4106.2 | 20.4 |
| 85 | 7/9/2015 | 7/10/2015 | 7 | 3.58 | 3578.7 | 22.3 |
| 86 | 7/10/2015 | 7/11/2015 | 5 | 3.65 | 3650.4 | 27.5 |
| 87 | 7/11/2015 | 7/12/2015 | 8 | 2.51 | 2506.4 | 14.8 |
| 88 | 7/12/2015 | 7/13/2015 | 5 | 4.91 | 4913.1 | 32.5 |
| 89 | 7/13/2015 | 7/14/2015 | 9 | 2.48 | 2483.9 | 16.5 |
| 90 | 7/14/2015 | 7/15/2015 | 5 | 3.17 | 3167.9 | 23.3 |
| 91 | 7/15/2015 | 7/16/2015 | 5 | 2.70 | 2702.0 | 22.0 |
| 92 | 7/16/2015 | 7/17/2015 | 5 | 10.19 | 10189.5 | 58.7 |
| 93 | 7/17/2015 | 7/18/2015 | 8 | 13.18 | 13175.2 | 38.2 |
| 94 | 7/18/2015 | 7/19/2015 | 5 | 6.43 | 6434.2 | 41.6 |
| 95 | 7/19/2015 | 7/20/2015 | 8 | 7.81 | 7813.7 | 41.7 |
| 96 | 7/20/2015 | 7/21/2015 | 5 | 1.69 | 1690.9 | 21.9 |
| 97 | 7/21/2015 | 7/22/2015 | 8 | 3.62 | 3621.3 | 19.5 |
| 98 | 7/22/2015 | 7/23/2015 | 5 | 2.52 | 2515.4 | 20.7 |
| 99 | 7/23/2015 | 7/24/2015 | 7 | 2.72 | 2718.6 | 19.3 |
| 100 | 7/24/2015 | 7/25/2015 | 5 | 2.83 | 2833.6 | 21.5 |
| 101 | 7/25/2015 | 7/26/2015 | 8 | 3.23 | 3232.7 | 14.8 |
| 102 | 7/26/2015 | 7/27/2015 | 5 | 1.93 | 1932.0 | 22.5 |
| 103 | 7/27/2015 | 7/28/2015 | 7 | 3.11 | 3109.0 | 19.9 |
| 104 | 7/28/2015 | 7/29/2015 | 5 | 3.03 | 3031.0 | 20.5 |
| 105 | 7/29/2015 | 7/30/2015 | 9 | 2.66 | 2663.7 | 12.6 |
| 106 | 7/30/2015 | 7/31/2015 | 5 | 3.74 | 3736.5 | 21.5 |
| 107 | 7/31/2015 | 8/1/2015 | 7 | 2.26 | 2258.2 | 12.8 |
| 108 | 8/1/2015 | 8/2/2015 | 5 | 1.46 | 1460.1 | 12.9 |
| 109 | 8/2/2015 | 8/3/2015 | 7 | 2.84 | 2838.9 | 20.0 |
| 110 | 8/3/2015 | 8/4/2015 | 5 | 5.37 | 5368.9 | 34.0 |
| 111 | 8/4/2015 | 8/5/2015 | 7 | 12.35 | 12350.1 | 45.8 |
| 112 | 8/5/2015 | 8/6/2015 | 5 | 2.66 | 2663.6 | 23.2 |
| 113 | 8/6/2015 | 8/7/2015 | 6 | 3.85 | 3850.9 | 20.2 |
| 114 | 8/11/2015 | 8/12/2015 | 3 | 2.06 | 2057.5 | 29.5 |
| 115 | 8/12/2015 | 8/13/2015 | 5 | 1.60 | 1597.0 | 15.8 |
| 116 | 8/13/2015 | 8/14/2015 | 5 | 2.50 | 2497.8 | 18.0 |
| 117 | 8/14/2015 | 8/15/2015 | 7 | 3.21 | 3208.6 | 23.1 |
| 118 | 8/15/2015 | 8/16/2015 | 5 | 4.20 | 4195.7 | 27.3 |
| 119 | 8/16/2015 | 8/17/2015 | 7 | 5.62 | 5621.1 | 29.8 |
| 120 | 8/17/2015 | 8/18/2015 | 5 | 2.93 | 2932.1 | 19.6 |
| 121 | 8/18/2015 | 8/19/2015 | 6 | 12.39 | 12385.7 | 54.7 |
| 122 | 8/19/2015 | 8/20/2015 | 5 | 1.58 | 1578.1 | 17.4 |
| 123 | 8/20/2015 | 8/21/2015 | 8 | 16.26 | 16262.5 | 53.5 |
| 124 | 8/21/2015 | 8/22/2015 | 5 | 6.47 | 6468.8 | 33.3 |
| 125 | 8/22/2015 | 8/23/2015 | 7 | 3.32 | 3320.8 | 21.6 |
| 126 | 8/23/2015 | 8/24/2015 | 5 | 3.89 | 3888.7 | 28.6 |
| 127 | 8/24/2015 | 8/25/2015 | 9 | 9.73 | 9733.9 | 38.9 |
| 128 | 8/25/2015 | 8/26/2015 | 5 | 1.26 | 1261.6 | 14.2 |
| 129 | 8/26/2015 | 8/27/2015 | 7 | 6.67 | 6674.5 | 28.6 |
| 130 | 8/27/2015 | 8/28/2015 | 5 | 6.16 | 6162.7 | 41.4 |
| 131 | 8/28/2015 | 8/29/2015 | 7 | 4.02 | 4022.7 | 20.0 |
| 132 | 8/29/2015 | 8/30/2015 | 5 | 1.56 | 1558.2 | 20.4 |
| 133 | 8/30/2015 | 8/31/2015 | 7 | 3.04 | 3038.7 | 19.4 |
| 134 | 8/31/2015 | 9/1/2015 | 5 | 3.92 | 3923.0 | 25.7 |
| 135 | 9/1/2015 | 9/2/2015 | 6 | 4.50 | 4496.4 | 27.3 |
| 136 | 9/2/2015 | 9/3/2015 | 5 | 6.11 | 6106.7 | 39.8 |
| 137 | 9/3/2015 | 9/4/2015 | 9 | 11.93 | 11932.2 | 38.2 |
| 138 | 9/4/2015 | 9/5/2015 | 5 | 10.04 | 10042.2 | 43.4 |
| 139 | 9/5/2015 | 9/6/2015 | 7 | 3.78 | 3776.7 | 30.3 |
| 140 | 9/6/2015 | 9/7/2015 | 5 | 8.86 | 8863.1 | 58.1 |
| 141 | 9/7/2015 | 9/8/2015 | 7 | 13.81 | 13807.8 | 54.1 |
| 142 | 9/8/2015 | 9/9/2015 | 5 | 1.73 | 1732.4 | 20.5 |
| 143 | 9/9/2015 | 9/10/2015 | 9 | 0.79 | 791.5 | 8.7 |
| 144 | 9/10/2015 | 9/11/2015 | 5 | 2.34 | 2335.9 | 30.3 |
| 145 | 9/11/2015 | 9/12/2015 | 6 | 2.03 | 2027.9 | 14.8 |
| 146 | 9/12/2015 | 9/13/2015 | 5 | 2.22 | 2216.7 | 24.7 |
| 147 | 9/13/2015 | 9/14/2015 | 8 | 6.91 | 6907.4 | 37.9 |
| 148 | 9/14/2015 | 9/15/2015 | 5 | 9.40 | 9398.7 | 43.1 |
| 149 | 9/15/2015 | 9/16/2015 | 10 | 12.21 | 12211.8 | 45.6 |
| 150 | 9/16/2015 | 9/17/2015 | 5 | 0.77 | 769.7 | 10.7 |
| 151 | 9/17/2015 | 9/18/2015 | 7 | 1.71 | 1712.2 | 12.2 |
| 152 | 9/18/2015 | 9/19/2015 | 5 | 0.31 | 308.2 | 8.7 |
| 153 | 9/19/2015 | 9/20/2015 | 7 | 15.89 | 15887.8 | 57.2 |
| 154 | 9/20/2015 | 9/21/2015 | 5 | 13.48 | 13483.6 | 50.9 |
| 155 | 9/21/2015 | 9/22/2015 | 7 | 8.10 | 8100.3 | 36.7 |
| 156 | 9/22/2015 | 9/23/2015 | 5 | 4.31 | 4313.7 | 40.4 |
| 157 | 9/23/2015 | 9/24/2015 | 8 | 16.35 | 16353.9 | 64.1 |
| 158 | 9/24/2015 | 9/25/2015 | 5 | 5.02 | 5015.0 | 42.4 |
| 159 | 9/25/2015 | 9/26/2015 | 7 | 7.73 | 7727.1 | 35.6 |
| 160 | 9/26/2015 | 9/27/2015 | 5 | 5.36 | 5360.0 | 47.6 |
| 161 | 9/27/2015 | 9/28/2015 | 6 | 7.90 | 7899.4 | 38.7 |
| 162 | 9/28/2015 | 9/29/2015 | 5 | 1.07 | 1069.1 | 17.1 |
| 163 | 9/29/2015 | 9/30/2015 | 9 | 2.25 | 2253.6 | 17.4 |
| 164 | 9/30/2015 | 10/1/2015 | 5 | 2.53 | 2531.9 | 32.0 |
| 165 | 10/1/2015 | 10/2/2015 | 6 | 7.38 | 7377.7 | 47.1 |
| 166 | 10/2/2015 | 10/3/2015 | 5 | 2.45 | 2449.3 | 28.8 |
| 167 | 10/3/2015 | 10/4/2015 | 7 | 2.49 | 2488.3 | 19.0 |
| 168 | 10/4/2015 | 10/5/2015 | 5 | 0.17 | 171.9 | 4.7 |
| 169 | 10/5/2015 | 10/6/2015 | 5 | 1.00 | 995.4 | 11.0 |
| 170 | 10/6/2015 | 10/7/2015 | 5 | 2.91 | 2908.9 | 26.1 |
| 172 | 10/8/2015 | 10/9/2015 | 2 | 1.23 | 1231.1 | 28.9 |
| 173 | 10/9/2015 | 10/10/2015 | 5 | 2.58 | 2584.7 | 25.8 |
| 174 | 10/10/2015 | 10/11/2015 | 5 | 4.86 | 4856.5 | 29.4 |
| 175 | 10/11/2015 | 10/12/2015 | 7 | 4.58 | 4579.9 | 28.9 |
| 176 | 10/12/2015 | 10/13/2015 | 5 | 1.85 | 1845.8 | 18.9 |
| 177 | 10/13/2015 | 10/14/2015 | 8 | 11.49 | 11488.4 | 46.0 |
| 178 | 10/14/2015 | 10/15/2015 | 5 | 3.38 | 3379.4 | 35.0 |
| 179 | 10/15/2015 | 10/16/2015 | 8 | 7.82 | 7818.2 | 38.0 |
| 180 | 10/16/2015 | 10/17/2015 | 5 | 3.35 | 3353.6 | 31.4 |
| 181 | 10/17/2015 | 10/18/2015 | 7 | 3.66 | 3659.4 | 21.6 |
| 182 | 10/18/2015 | 10/19/2015 | 5 | 2.88 | 2877.1 | 26.6 |
| 183 | 10/19/2015 | 10/20/2015 | 6 | 3.08 | 3076.8 | 20.2 |
| 184 | 10/20/2015 | 10/21/2015 | 5 | 1.41 | 1408.1 | 16.4 |
| 185 | 10/21/2015 | 10/22/2015 | 6 | 2.48 | 2477.0 | 17.9 |
| 186 | 10/22/2015 | 10/23/2015 | 5 | 1.55 | 1548.9 | 19.9 |
| 187 | 10/23/2015 | 10/24/2015 | 7 | 2.08 | 2084.8 | 15.3 |
| 188 | 10/24/2015 | 10/25/2015 | 5 | 3.32 | 3324.0 | 30.2 |
| 189 | 10/25/2015 | 10/26/2015 | 7 | 2.77 | 2770.4 | 21.2 |
| 190 | 10/26/2015 | 10/27/2015 | 5 | 6.08 | 6083.7 | 39.8 |
| 191 | 10/27/2015 | 10/28/2015 | 7 | 3.00 | 2995.2 | 17.8 |
| 192 | 10/28/2015 | 10/29/2015 | 5 | 0.51 | 508.6 | 7.8 |
| 193 | 10/29/2015 | 10/30/2015 | 6 | 7.73 | 7733.8 | 39.1 |
| 195 | 11/10/2015 | 11/11/2015 | 5 | 3.25 | 3250.7 | 25.7 |
| 196 | 11/11/2015 | 11/12/2015 | 5 | 3.82 | 3817.8 | 29.0 |
| 197 | 11/12/2015 | 11/13/2015 | 7 | 2.36 | 2363.2 | 19.2 |
| 198 | 11/13/2015 | 11/14/2015 | 5 | 1.99 | 1994.0 | 18.0 |
| 199 | 11/14/2015 | 11/15/2015 | 6 | 3.70 | 3698.6 | 25.1 |
| 200 | 11/15/2015 | 11/16/2015 | 5 | 4.62 | 4621.0 | 38.6 |
| 201 | 11/16/2015 | 11/17/2015 | 7 | 0.92 | 923.5 | 10.0 |
| 202 | 11/17/2015 | 11/18/2015 | 5 | 1.93 | 1931.4 | 18.2 |
| 203 | 11/18/2015 | 11/19/2015 | 7 | 1.26 | 1257.3 | 11.9 |
| 204 | 11/19/2015 | 11/20/2015 | 5 | 1.54 | 1539.6 | 22.4 |
| 205 | 11/20/2015 | 11/21/2015 | 10 | 3.34 | 3335.7 | 18.7 |
| 206 | 11/21/2015 | 11/22/2015 | 5 | 0.29 | 288.1 | 7.1 |
| 207 | 11/22/2015 | 11/23/2015 | 6 | 1.87 | 1874.7 | 17.1 |
| 208 | 11/23/2015 | 11/24/2015 | 5 | 0.49 | 488.8 | 7.8 |
| 209 | 11/24/2015 | 11/25/2015 | 9 | 2.32 | 2318.0 | 14.0 |
| 210 | 11/25/2015 | 11/26/2015 | 5 | 1.77 | 1770.4 | 17.0 |
| 211 | 11/26/2015 | 11/27/2015 | 6 | 6.14 | 6143.9 | 32.4 |
| 212 | 11/27/2015 | 11/28/2015 | 5 | 0.87 | 870.3 | 10.2 |
| 213 | 11/28/2015 | 11/29/2015 | 7 | 4.61 | 4611.8 | 27.6 |
| 214 | 11/29/2015 | 11/30/2015 | 5 | 1.47 | 1473.0 | 20.1 |
| 215 | 11/30/2015 | 12/1/2015 | 7 | 7.94 | 7939.2 | 41.5 |
| 216 | 12/1/2015 | 12/2/2015 | 5 | 8.67 | 8670.8 | 49.8 |
| 217 | 12/2/2015 | 12/3/2015 | 8 | 10.65 | 10647.5 | 54.3 |
| 218 | 12/3/2015 | 12/4/2015 | 5 | 21.24 | 21235.9 | 96.4 |
| 219 | 12/4/2015 | 12/5/2015 | 7 | 10.41 | 10407.6 | 46.5 |
| 220 | 12/5/2015 | 12/6/2015 | 5 | 23.28 | 23275.6 | 79.8 |
| 221 | 12/6/2015 | 12/7/2015 | 6 | 1.76 | 1756.8 | 16.6 |
| 222 | 12/7/2015 | 12/8/2015 | 5 | 12.74 | 12742.1 | 62.7 |
| 223 | 12/8/2015 | 12/9/2015 | 7 | 2.17 | 2171.0 | 12.8 |
| 224 | 12/9/2015 | 12/10/2015 | 5 | 5.00 | 4995.7 | 45.2 |
| 225 | 12/10/2015 | 12/11/2015 | 8 | 2.14 | 2140.6 | 13.2 |
| 226 | 12/11/2015 | 12/12/2015 | 5 | 6.68 | 6675.3 | 47.0 |
| 227 | 12/12/2015 | 12/13/2015 | 7 | 1.65 | 1645.0 | 13.8 |
| 228 | 12/13/2015 | 12/14/2015 | 5 | 0.24 | 242.7 | 5.3 |
| 229 | 12/14/2015 | 12/15/2015 | 7 | 1.44 | 1437.6 | 13.0 |
| 230 | 12/15/2015 | 12/16/2015 | 5 | 0.52 | 525.0 | 10.5 |
| 231 | 12/16/2015 | 12/17/2015 | 5 | 0.41 | 406.9 | 10.1 |
| 232 | 12/17/2015 | 12/18/2015 | 5 | 0.62 | 619.8 | 9.8 |
| 233 | 12/18/2015 | 12/19/2015 | 8 | 5.64 | 5641.4 | 28.2 |
| 234 | 12/19/2015 | 12/20/2015 | 5 | 2.89 | 2886.4 | 33.9 |
| 235 | 12/20/2015 | 12/21/2015 | 7 | 3.82 | 3820.2 | 22.7 |
| 236 | 12/21/2015 | 12/22/2015 | 5 | 3.28 | 3282.3 | 23.1 |
| 237 | 12/22/2015 | 12/23/2015 | 6 | 4.76 | 4763.9 | 40.4 |
| 238 | 12/23/2015 | 12/24/2015 | 5 | 1.22 | 1216.6 | 17.7 |
| 239 | 12/24/2015 | 12/25/2015 | 7 | 3.59 | 3585.6 | 24.2 |
| 240 | 12/25/2015 | 12/26/2015 | 5 | 1.82 | 1817.6 | 17.4 |
| 241 | 12/26/2015 | 12/27/2015 | 9 | 14.15 | 14146.9 | 39.8 |
| 242 | 12/27/2015 | 12/28/2015 | 5 | 8.28 | 8279.2 | 46.7 |
| 243 | 12/28/2015 | 12/29/2015 | 7 | 12.44 | 12441.3 | 50.1 |
| 244 | 12/29/2015 | 12/30/2015 | 5 | 2.35 | 2352.0 | 22.5 |
| 245 | 12/30/2015 | 12/31/2015 | 7 | 27.33 | 27325.2 | 86.9 |
| 246 | 12/31/2015 | 1/1/2016 | 5 | 3.72 | 3716.4 | 26.2 |
| 247 | 1/1/2016 | 1/2/2016 | 5 | 1.08 | 1081.5 | 11.4 |
| 248 | 1/2/2016 | 1/3/2016 | 5 | 1.20 | 1200.3 | 13.5 |
| 249 | 1/3/2016 | 1/4/2016 | 6 | 7.63 | 7630.8 | 42.7 |
| 250 | 1/4/2016 | 1/5/2016 | 5 | 1.72 | 1722.0 | 17.1 |
| 251 | 1/5/2016 | 1/6/2016 | 8 | 4.53 | 4525.9 | 24.1 |
| 252 | 1/6/2016 | 1/7/2016 | 5 | 1.95 | 1952.0 | 20.4 |
| 253 | 1/7/2016 | 1/8/2016 | 8 | 7.72 | 7717.9 | 34.5 |
| 254 | 1/8/2016 | 1/9/2016 | 5 | 2.72 | 2725.0 | 31.5 |
| 255 | 1/9/2016 | 1/10/2016 | 5 | 1.22 | 1217.1 | 14.5 |
| 256 | 1/10/2016 | 1/11/2016 | 5 | 0.93 | 925.3 | 12.3 |
| 257 | 1/11/2016 | 1/12/2016 | 6 | 1.62 | 1620.8 | 13.8 |
| 258 | 1/12/2016 | 1/13/2016 | 5 | 4.78 | 4784.3 | 30.1 |
| 259 | 1/13/2016 | 1/14/2016 | 6 | 2.68 | 2684.4 | 22.5 |
| 260 | 1/14/2016 | 1/15/2016 | 5 | 1.59 | 1593.6 | 23.9 |
| 261 | 1/15/2016 | 1/16/2016 | 7 | 4.25 | 4252.7 | 31.2 |
| 262 | 1/16/2016 | 1/17/2016 | 5 | 2.17 | 2172.7 | 23.8 |
| 263 | 1/17/2016 | 1/18/2016 | 7 | 2.51 | 2505.8 | 21.2 |
| 264 | 1/18/2016 | 1/19/2016 | 5 | 4.90 | 4901.7 | 41.0 |
| 265 | 1/19/2016 | 1/20/2016 | 5 | 3.40 | 3404.9 | 33.1 |
| 266 | 1/20/2016 | 1/21/2016 | 5 | 1.95 | 1946.9 | 18.2 |
| 267 | 1/21/2016 | 1/22/2016 | 7 | 3.88 | 3882.6 | 20.9 |
| 268 | 1/22/2016 | 1/23/2016 | 5 | 1.29 | 1289.0 | 13.9 |
| 269 | 1/23/2016 | 1/24/2016 | 7 | 6.86 | 6861.5 | 34.4 |
| 270 | 1/24/2016 | 1/25/2016 | 5 | 4.87 | 4866.2 | 39.0 |
| 271 | 1/25/2016 | 1/26/2016 | 7 | 3.78 | 3776.4 | 18.4 |
| 272 | 1/26/2016 | 1/27/2016 | 5 | 2.19 | 2187.3 | 18.6 |
| 273 | 1/27/2016 | 1/28/2016 | 7 | 1.53 | 1532.6 | 13.0 |
| 274 | 1/28/2016 | 1/29/2016 | 5 | 4.16 | 4158.4 | 26.0 |
| 275 | 1/29/2016 | 1/30/2016 | 5 | 22.49 | 22488.5 | 66.0 |
| 276 | 1/30/2016 | 1/31/2016 | 5 | 18.86 | 18857.6 | 90.7 |
| 277 | 1/31/2016 | 2/1/2016 | 5 | 7.20 | 7203.2 | 37.3 |
| 278 | 2/1/2016 | 2/2/2016 | 5 | 28.12 | 28120.0 | 86.5 |
| 279 | 2/2/2016 | 2/3/2016 | 5 | 0.32 | 324.4 | 8.5 |
| 280 | 2/3/2016 | 2/4/2016 | 5 | 0.65 | 652.8 | 11.8 |
| 281 | 2/4/2016 | 2/5/2016 | 7 | 1.90 | 1900.9 | 14.4 |
| 282 | 2/5/2016 | 2/6/2016 | 5 | 1.03 | 1034.4 | 14.1 |
| 283 | 2/6/2016 | 2/7/2016 | 6 | 2.52 | 2523.3 | 19.3 |
| 284 | 2/7/2016 | 2/8/2016 | 5 | 1.20 | 1202.0 | 13.1 |
| 285 | 2/8/2016 | 2/9/2016 | 6 | 2.92 | 2920.6 | 19.7 |
| 286 | 2/9/2016 | 2/10/2016 | 5 | 2.50 | 2495.2 | 22.6 |
| 287 | 2/10/2016 | 2/11/2016 | 6 | 1.24 | 1243.6 | 10.5 |
| 288 | 2/11/2016 | 2/12/2016 | 5 | 0.93 | 932.7 | 13.6 |
| 289 | 2/12/2016 | 2/13/2016 | 6 | 3.56 | 3560.3 | 22.3 |
| 290 | 2/13/2016 | 2/14/2016 | 5 | 1.01 | 1012.9 | 13.6 |
| 291 | 2/14/2016 | 2/15/2016 | 7 | 3.23 | 3232.7 | 18.5 |
| 292 | 2/15/2016 | 2/16/2016 | 5 | 1.46 | 1456.6 | 14.4 |
| 293 | 2/16/2016 | 2/17/2016 | 7 | 2.05 | 2047.2 | 17.8 |
| 294 | 2/17/2016 | 2/18/2016 | 5 | 1.20 | 1200.9 | 16.5 |
| 295 | 2/18/2016 | 2/19/2016 | 6 | 8.57 | 8565.1 | 39.0 |
| 296 | 2/19/2016 | 2/20/2016 | 5 | 0.82 | 819.6 | 13.8 |
| 297 | 2/20/2016 | 2/21/2016 | 6 | 2.25 | 2253.6 | 13.9 |
| 298 | 2/21/2016 | 2/22/2016 | 5 | 0.72 | 719.8 | 9.2 |
| 299 | 2/22/2016 | 2/23/2016 | 6 | 2.38 | 2377.7 | 16.0 |
| 300 | 2/23/2016 | 2/24/2016 | 5 | 8.75 | 8745.4 | 56.4 |
| 301 | 2/24/2016 | 2/25/2016 | 6 | 5.96 | 5963.8 | 40.3 |
| 302 | 2/25/2016 | 2/26/2016 | 5 | 14.67 | 14666.6 | 76.8 |
| 303 | 2/26/2016 | 2/27/2016 | 6 | 10.28 | 10280.4 | 59.7 |
| 304 | 2/27/2016 | 2/28/2016 | 5 | 14.25 | 14251.7 | 62.4 |
| 305 | 2/28/2016 | 2/29/2016 | 7 | 18.09 | 18092.5 | 64.4 |
| 306 | 2/29/2016 | 3/1/2016 | 5 | 23.32 | 23317.6 | 75.1 |
| 307 | 3/1/2016 | 3/2/2016 | 7 | 1.61 | 1607.1 | 14.2 |
| 308 | 3/2/2016 | 3/3/2016 | 5 | 4.98 | 4978.8 | 33.1 |
| 309 | 3/3/2016 | 3/4/2016 | 6 | 1.72 | 1720.5 | 15.2 |
| 310 | 3/4/2016 | 3/5/2016 | 5 | 4.60 | 4598.1 | 34.4 |
| 311 | 3/5/2016 | 3/6/2016 | 7 | 3.62 | 3623.6 | 21.2 |
| 312 | 3/6/2016 | 3/7/2016 | 5 | 1.47 | 1468.1 | 17.3 |
| 313 | 3/7/2016 | 3/8/2016 | 6 | 3.57 | 3572.4 | 18.5 |
| 314 | 3/8/2016 | 3/9/2016 | 5 | 2.00 | 1998.1 | 18.3 |
| 315 | 3/9/2016 | 3/10/2016 | 7 | 3.63 | 3630.8 | 28.1 |
| 316 | 3/10/2016 | 3/11/2016 | 5 | 8.84 | 8835.9 | 39.6 |
| 317 | 3/11/2016 | 3/12/2016 | 7 | 1.18 | 1177.7 | 10.9 |
| 318 | 3/12/2016 | 3/13/2016 | 5 | 4.89 | 4892.4 | 32.8 |
| 319 | 3/13/2016 | 3/14/2016 | 5 | 4.08 | 4080.0 | 35.5 |
| 320 | 3/14/2016 | 3/15/2016 | 5 | 3.55 | 3546.8 | 27.7 |
| 321 | 3/15/2016 | 3/16/2016 | 7 | 1.16 | 1155.4 | 11.0 |
| 322 | 3/16/2016 | 3/17/2016 | 5 | 6.41 | 6407.8 | 48.7 |
| 323 | 3/17/2016 | 3/18/2016 | 8 | 6.10 | 6103.9 | 33.2 |
| 324 | 3/18/2016 | 3/19/2016 | 5 | 5.85 | 5849.4 | 43.7 |
| 325 | 3/19/2016 | 3/20/2016 | 6 | 2.41 | 2412.0 | 18.3 |
| 326 | 3/20/2016 | 3/21/2016 | 5 | 1.93 | 1932.8 | 22.5 |
| 327 | 3/21/2016 | 3/22/2016 | 7 | 4.06 | 4056.4 | 28.0 |
| 328 | 3/22/2016 | 3/23/2016 | 5 | 3.45 | 3451.2 | 31.5 |
| 329 | 3/23/2016 | 3/24/2016 | 6 | 5.38 | 5380.7 | 37.7 |
| 330 | 3/24/2016 | 3/25/2016 | 5 | 8.68 | 8681.8 | 48.7 |
| 331 | 3/25/2016 | 3/26/2016 | 8 | 16.07 | 16070.8 | 55.7 |
| 332 | 3/26/2016 | 3/27/2016 | 5 | 1.78 | 1779.3 | 15.9 |
| 333 | 3/27/2016 | 3/28/2016 | 7 | 2.78 | 2779.5 | 17.6 |
| 334 | 3/28/2016 | 3/29/2016 | 5 | 6.59 | 6591.7 | 48.4 |
| 335 | 3/29/2016 | 3/30/2016 | 6 | 8.06 | 8065.0 | 39.5 |
| 336 | 3/30/2016 | 3/31/2016 | 5 | 1.58 | 1581.4 | 17.1 |
| 337 | 3/31/2016 | 4/1/2016 | 8 | 6.34 | 6343.6 | 38.3 |
| 338 | 4/1/2016 | 4/2/2016 | 7 | 7.03 | 7028.6 | 36.5 |
| 339 | 4/2/2016 | 4/3/2016 | 5 | 4.71 | 4710.9 | 34.5 |
| 340 | 4/3/2016 | 4/4/2016 | 8 | 2.21 | 2211.6 | 15.1 |
| 341 | 4/4/2016 | 4/5/2016 | 5 | 1.72 | 1718.3 | 18.5 |
| 342 | 4/5/2016 | 4/6/2016 | 6 | 19.37 | 19369.0 | 61.5 |
| 343 | 4/6/2016 | 4/7/2016 | 5 | 19.48 | 19476.4 | 77.5 |
| 344 | 4/7/2016 | 4/8/2016 | 6 | 34.97 | 34965.6 | 107.5 |
| 345 | 4/8/2016 | 4/9/2016 | 2 | 0.69 | 694.5 | 22.2 |
| 346 | 4/9/2016 | 4/10/2016 | 6 | 2.95 | 2951.2 | 22.9 |
| 347 | 4/10/2016 | 4/11/2016 | 5 | 1.59 | 1587.1 | 14.7 |
| 348 | 4/11/2016 | 4/12/2016 | 8 | 4.15 | 4149.4 | 14.7 |
| 349 | 4/12/2016 | 4/13/2016 | 5 | 2.00 | 2003.8 | 17.8 |
| 350 | 4/13/2016 | 4/14/2016 | 7 | 5.04 | 5039.4 | 24.0 |
| 351 | 4/14/2016 | 4/15/2016 | 5 | 1.95 | 1953.4 | 18.4 |
| 352 | 4/15/2016 | 4/16/2016 | 8 | 5.27 | 5274.1 | 23.5 |
| 353 | 4/16/2016 | 4/17/2016 | 5 | 2.37 | 2369.3 | 19.6 |
| 354 | 4/17/2016 | 4/18/2016 | 7 | 8.75 | 8754.3 | 35.8 |
| 355 | 4/18/2016 | 4/19/2016 | 5 | 1.32 | 1323.7 | 18.9 |
| 356 | 4/19/2016 | 4/20/2016 | 11 | 5.30 | 5304.1 | 26.6 |
| 357 | 4/20/2016 | 4/21/2016 | 5 | 5.60 | 5598.9 | 31.2 |
| 358 | 4/21/2016 | 4/22/2016 | 6 | 1.90 | 1898.4 | 22.4 |
| 359 | 4/22/2016 | 4/23/2016 | 5 | 3.38 | 3382.6 | 33.6 |
| 360 | 4/23/2016 | 4/24/2016 | 8 | 5.91 | 5905.6 | 32.3 |
| 361 | 4/24/2016 | 4/25/2016 | 5 | 5.27 | 5270.7 | 31.6 |
| 362 | 4/25/2016 | 4/26/2016 | 7 | 2.67 | 2673.6 | 22.0 |
| 363 | 4/26/2016 | 4/27/2016 | 5 | 1.27 | 1268.4 | 14.5 |
| 364 | 4/27/2016 | 4/28/2016 | 8 | 7.11 | 7113.9 | 28.8 |
| 365 | 4/28/2016 | 4/29/2016 | 5 | 4.99 | 4991.2 | 35.8 |
| 366 | 4/29/2016 | 4/30/2016 | 7 | 1.32 | 1318.8 | 12.5 |
| 367 | 4/30/2016 | 5/1/2016 | 5 | 1.64 | 1636.9 | 13.8 |
| 368 | 5/1/2016 | 5/2/2016 | 7 | 2.74 | 2736.2 | 18.0 |
| 369 | 5/2/2016 | 5/3/2016 | 5 | 2.75 | 2748.1 | 20.2 |
| 370 | 5/3/2016 | 5/4/2016 | 8 | 4.41 | 4409.9 | 19.7 |
| 371 | 5/4/2016 | 5/5/2016 | 5 | 4.12 | 4122.5 | 31.6 |
| 372 | 5/5/2016 | 5/6/2016 | 7 | 5.47 | 5465.6 | 26.6 |
| 373 | 5/6/2016 | 5/7/2016 | 5 | 4.85 | 4850.0 | 31.9 |
| 374 | 5/7/2016 | 5/8/2016 | 5 | 4.26 | 4264.1 | 31.4 |
| 375 | 5/8/2016 | 5/9/2016 | 5 | 3.02 | 3023.9 | 30.5 |
| 376 | 5/9/2016 | 5/10/2016 | 7 | 3.54 | 3536.5 | 26.8 |
| 377 | 5/10/2016 | 5/11/2016 | 5 | 2.58 | 2580.2 | 21.6 |
| 378 | 5/11/2016 | 5/12/2016 | 5 | 3.37 | 3371.7 | 26.3 |
| 379 | 5/12/2016 | 5/13/2016 | 5 | 1.85 | 1852.8 | 21.4 |
| 380 | 5/13/2016 | 5/14/2016 | 7 | 5.23 | 5228.4 | 30.7 |
| 381 | 5/14/2016 | 5/15/2016 | 5 | 8.33 | 8325.0 | 49.7 |
| 382 | 5/15/2016 | 5/16/2016 | 7 | 4.45 | 4454.9 | 25.6 |
| 383 | 5/16/2016 | 5/17/2016 | 5 | 4.10 | 4100.0 | 27.8 |
| 384 | 5/17/2016 | 5/18/2016 | 10 | 5.65 | 5653.7 | 22.5 |
| 385 | 5/18/2016 | 5/19/2016 | 5 | 1.45 | 1453.1 | 17.3 |
| 386 | 5/19/2016 | 5/20/2016 | 6 | 2.58 | 2584.4 | 17.8 |
| 387 | 5/20/2016 | 5/21/2016 | 5 | 1.91 | 1911.8 | 18.3 |
| 388 | 5/21/2016 | 5/22/2016 | 8 | 2.37 | 2366.8 | 15.5 |
| 389 | 5/22/2016 | 5/23/2016 | 5 | 1.38 | 1380.1 | 18.6 |
| 390 | 5/23/2016 | 5/24/2016 | 6 | 1.65 | 1651.8 | 12.5 |
| 391 | 5/24/2016 | 5/25/2016 | 5 | 3.75 | 3753.3 | 24.8 |
| 392 | 5/25/2016 | 5/26/2016 | 6 | 4.40 | 4397.8 | 30.0 |
| 393 | 5/26/2016 | 5/27/2016 | 5 | 3.84 | 3837.0 | 32.9 |
| 394 | 5/27/2016 | 5/28/2016 | 8 | 5.22 | 5216.7 | 26.5 |
| 395 | 5/28/2016 | 5/29/2016 | 5 | 3.69 | 3694.7 | 28.7 |
| 396 | 5/29/2016 | 5/30/2016 | 5 | 3.18 | 3179.3 | 25.3 |
| 397 | 5/30/2016 | 5/31/2016 | 5 | 4.87 | 4873.2 | 32.1 |
| 398 | 5/31/2016 | 6/1/2016 | 7 | 22.63 | 22625.2 | 83.3 |
| 399 | 6/1/2016 | 6/2/2016 | 5 | 2.54 | 2539.8 | 22.9 |
| 400 | 6/2/2016 | 6/3/2016 | 8 | 19.24 | 19241.3 | 75.8 |
| 401 | 6/3/2016 | 6/4/2016 | 5 | 27.29 | 27293.3 | 72.8 |
| 402 | 6/4/2016 | 6/5/2016 | 6 | 8.07 | 8071.1 | 42.8 |
| 403 | 6/5/2016 | 6/6/2016 | 5 | 8.06 | 8061.0 | 43.9 |
| 404 | 6/6/2016 | 6/7/2016 | 9 | 17.17 | 17171.5 | 44.5 |
| 405 | 6/7/2016 | 6/8/2016 | 5 | 9.15 | 9146.8 | 60.0 |
| 406 | 6/8/2016 | 6/9/2016 | 6 | 23.96 | 23956.3 | 69.4 |
| 407 | 6/9/2016 | 6/10/2016 | 5 | 1.05 | 1046.5 | 15.7 |
| 408 | 6/10/2016 | 6/11/2016 | 6 | 5.23 | 5233.9 | 30.4 |
| 409 | 6/11/2016 | 6/12/2016 | 5 | 9.32 | 9317.7 | 44.7 |
| 410 | 6/12/2016 | 6/13/2016 | 7 | 6.70 | 6704.4 | 28.8 |
| 411 | 6/13/2016 | 6/14/2016 | 5 | 4.95 | 4948.1 | 24.7 |
| 412 | 6/14/2016 | 6/15/2016 | 5 | 6.82 | 6818.9 | 25.7 |
|  |  |  | **Average** | **5.39** | **5386.98** | **29.38** |

**Seasonal variation in daily movement of Great Indian Bustard**

| Season | mean | sd | max | min | Sum | median | Max in km |
| --- | --- | --- | --- | --- | --- | --- | --- |
| Post Monsoon | 3.15 | 2.32 | 11.49 | 0.17 | 154.59 | 2.58 | 11.49 |
| Monsoon | 5.00 | 3.89 | 16.35 | 0.31 | 440.15 | 3.60 | 16.35 |
| Winter | 5.39 | 6.20 | 28.12 | 0.24 | 485.14 | 2.81 | 28.12 |
| Summer | 6.17 | 6.37 | 38.99 | 0.26 | 1128.79 | 4.41 | 38.99 |

**R code to calculate daily movement of Great Indian Bustard**

library("adehabitatLT")

library("lubridate")

library("raster")

library("rworldmap")

library("sp")

library(tidyverse)

library(sf)

library(mapview)

library(move)

library(amt)

library(knitr)

library(lubridate)

library(maptools)

library(raster)

library(move)

library(amt)

library(ggmap)

library(tibble)

library(leaflet)

library(dplyr)

setwd("D:/Study_Material/Ongoing paper work/GIB_Movement_WileyE&E/GIB_WE&E ")

GIB <-read.csv(file.choose(), header=T)

head(GIB)

GIB$timestamp <- as.POSIXct(lubridate::mdy(GIB$date) +

lubridate::hms(GIB$time))

class(GIB)

GIB <- GIB[!is.na(GIB$X),]

GIB <- GIB[!is.na(GIB$Y),]

coordinates(GIB) <- c("X", "Y")

class(GIB)

proj4string(GIB) <- CRS("+init=epsg:32644")

class(GIB$GPSFixTime)

GIB$timestamp <- as.POSIXct(lubridate::mdy(GIB$date) +

lubridate::hms(GIB$time))

class(GIB$timestamp)

timediff <- diff(GIB$timestamp)

GIB <-GIB[-1,]

GIB$timediff <-as.numeric(abs(timediff))

str(GIB)

head(GIB)

summary(GIB$timediff)

GIB1 <- as.ltraj(coordinates(GIB),date=GIB$timestamp,id=GIB$id,

typeII=TRUE)

is.regular(GIB1)

GIB1

plot.ltraj(GIB1)

GIB1

head(GIB1[[1]])

plotltr(GIB1, which="dist")

plotltr(GIB1, which="dt")

plotltr(GIB1, which="R2n")

hist(GIB1[2], "dt", freq = TRUE)

hist(GIB1[2], "dist", freq = TRUE)

#convert ltraj to data.frame

GIB_df <- ld(GIB1)

head(GIB_df)

#parse date

Now we can isolate the date only and add that as the date column

GIB_df$date.utc <- as.Date(GIB_df$date, format="%Y-%m-%d")

GIB_df$time.utc <- format(GIB_df$date, format="%H:%M:%S")

write.csv(GIB_df," D:/Study_Material/Ongoing paper work/GIB_Movement_WileyE&E/GIB_WE&E ", row.names = FALSE)

#add local.date,local.time,longitude,latitute in newLT file

#delete the first row of added column

#amt_day-night

fisher.move <-read.csv(file.choose())

head(fisher.move)

# parse date and time and create time stamps

fisher.move$timestamp <- as.POSIXct(lubridate::dmy(fisher.move$date.utc) +

lubridate::hms(fisher.move$time.utc))

fisher.dat <- as(fisher.move, "data.frame")

ind <- complete.cases(fisher.dat[,c("longitude", "latitude", "timestamp")])

fisher.dat <- fisher.dat[ind == TRUE,]

ind2 <-fisher.dat %>% select(timestamp, longitude, latitude) %>%

duplicated

sum(ind2)

fisher.dat$timestamp<-as.POSIXct(fisher.dat$timestamp, format="%Y-%m-%d %H:%M:%0S", tz = "UTC")

trk <- mk_track(fisher.dat, .x=longitude, .y=latitude, .t=timestamp, id = id,

distance=dist, nsd=R2n, timediff=dt, TA=rel.angle, lmt.date=local.date, lmt.time=local.time,

crs = CRS("+init=epsg:4326"))

trk <- trk %>% time_of_day(include.crepuscule = FALSE)

write.csv(trk," D:/Study_Material/Ongoing paper work/GIB_Movement_WileyE&E/GIB_WE&E gib_all.csv", row.names = FALSE)

**R Code for Levy’s flight movement Analysis**

#1. Load your data

# Load libraries

library(geosphere) # For geographic distance (if using lat/lon)

library(fitdistrplus) # For fitting distributions

library(poweRlaw) # For power-law fitting

library(tidyverse)

setwd("//192.168.205.116/Shaheer_data/Wolf_Distribution/Layers2024/layers_to_use/newclipped")

# Load your data

data <- read.csv("//192.168.205.116/Shaheer_data/Ongoing paper work/GIB_Movement_WileyE&E/GIB_levy_mov.csv", header=T)

# If you have lat/lon, convert to distances

# For lat/lon, compute step lengths in meters:

data <- data %>%

arrange(fixtime) %>%

mutate(

lon_lead = lead(Long),

lat_lead = lead(Lat),

step_length = distHaversine(cbind(Long, Lat), cbind(lon_lead, lat_lead))

) %>%

select(-lon_lead, -lat_lead)

# If already in projected coordinates (x, y in meters), use:

data <- data %>%

arrange(sno.) %>%

mutate(

x_lead = lead(x),

y_lead = lead(y),

step_length = sqrt((x_lead - x)^2 + (y_lead - y)^2)

) %>%

select(-x_lead, -y_lead) # clean up temporary columns

# Plotting x and y from the dataframe

plot(data$x, data$y, type = "l", col = "black", xlab = "X", ylab = "Y", main = "Post Monsoon Movement Path")

#2. Remove zero-length or NA steps

data <- data %>% filter(!is.na(step_length) & step_length > 0)

#3. Fit Power-law (Lévy) distribution

# Create a power-law object

pl <- conpl$new(data$step_length)

# Estimate xmin (lower bound where power-law holds)

est <- estimate_xmin(pl)

pl$setXmin(est)

# Estimate alpha (scaling exponent ??)

est <- estimate_pars(pl)

pl$setPars(est)

# View estimated parameters

cat("Estimated xmin:", pl$getXmin(), "\n")

cat("Estimated ?? (alpha):", pl$pars, "\n")

#Estimate mu value to check for the levy flight movement pattern

est_pars <- estimate_pars(pl)

pl$setPars(est_pars)

pl$pars

# Plot the distribution

plot(pl, xlab = "Step Length", ylab = "Cumalative Fequency Distribution")

lines(pl, col = "red")

# Compare with exponential

exp <- conexp$new(data$step_length)

exp$setXmin(pl$getXmin())

exp$setPars(estimate_pars(exp))

compare_distributions <- compare_distributions(pl, exp)

print(compare_distributions)

#4. Visualizing Distribution

hist(data$step_length, breaks = 100, main = "Step Length Distribution", xlab = "Step length (m)", col = "lightblue", freq = TRUE)

curve(dexp(x, rate = 1/mean(data$step_length)), col = "red", add = TRUE)

#Optional: Truncated Power-Law (if you suspect a cutoff)

tpl <- conpl$new(data$step_length)

tpl$setXmin(est$xmin)

tpl$setPars(estimate_pars(tpl))

plot(tpl)

lines(tpl, col = "blue")

**R code for Directionality Analysis**

# Load required packages

library(geosphere) # For distance and bearing calculations

library(lubridate) # For date-time handling

library(dplyr) # For data wrangling

library(ggplot2) # For visualization

#run this if you want to convert utm into wgs projection for your data (line 9-35)

library(sf)

library(readr) # for reading csv

# Read CSV data

df <- read_csv("//192.168.205.116/Shaheer_data/Ongoing paper work/GIB_Movement_WileyE&E/GIB_levy_mov_winter.csv") # Replace with your actual CSV path

# Inspect column names

head(df)

# Convert to sf object (assuming UTM Zone 44N, EPSG:32644)

utm_sf <- st_as_sf(df, coords = c("x", "y"), crs = 32644)

# Transform to WGS84 (lat-long)

latlong_sf <- st_transform(utm_sf, crs = 4326)

# Extract coordinates

coords <- st_coordinates(latlong_sf)

# Add lat-long to original dataframe

df$Longitude <- coords[,1]

df$Latitude <- coords[,2]

# View updated data

head(df)

# Optional: Save new CSV

write_csv(df, "GIB_levy_mov_winter2.csv")

setwd("//192.168.205.116/Shaheer_data/Ongoing paper work/GIB_Movement_WileyE&E/1_Manuscript/Review/Review 2")

# Load your data

data <- read.csv("//192.168.205.116/Shaheer_data/Ongoing paper work/GIB_Movement_WileyE&E/GIBMovement.csv", header=T)

head(data)

# Format and sort the data

data <- data %>%

arrange(ID) %>%

mutate(Time_Field = mdy_hms(Time_Field))

# Calculate bearings between successive points

data <- data %>%

mutate(

lead_lat = lead(Lat),

lead_long = lead(Long),

bearing = bearing(cbind(Long, Lat), cbind(lead_long, lead_lat))

)

# Calculate turning angles

data <- data %>%

mutate(

prev_bearing = lag(bearing),

turning_angle = (bearing - prev_bearing + 360) %% 360,

turning_angle = ifelse(turning_angle > 180, 360 - turning_angle, turning_angle) # Normalize to [0, 180]

)

# Clean data

data_clean <- data %>% filter(!is.na(turning_angle))

# Summary of turning angles

summary(data_clean$turning_angle)

# Plot histogram

ggplot(data_clean, aes(x = turning_angle)) +

geom_histogram(binwidth = 10, fill = "gray", color = "black") +

labs(title = "Turning Angles of GIB Movement",

x = "Turning Angle (degrees)",

y = "Frequency") +

theme_minimal()

ggplot(data_clean, aes(x = turning_angle)) +

geom_histogram(binwidth = 10, fill = "skyblue", color = "black") +

coord_polar(start = 0) +

theme_minimal() +

labs(title = "Histogram of Turning Angles",

x = "Turning Angle (degrees)",

y = "Frequency") +

theme(

plot.title = element_text(size = 18, face = "bold"),

axis.title = element_text(size = 16),

axis.text = element_text(size = 14)

)
